# Supplementary figures and images for: Modulation of NMDA receptor signaling and zinc chelation prevent seizure-like events in a zebrafish model of SLC13A5 epilepsy
Source: PLoS Biol. 2025 Apr 10;23(4):e3002499. doi: 10.1371/journal.pbio.3002499 (PMC12047791; doi:10.1371/journal.pbio.3002499)

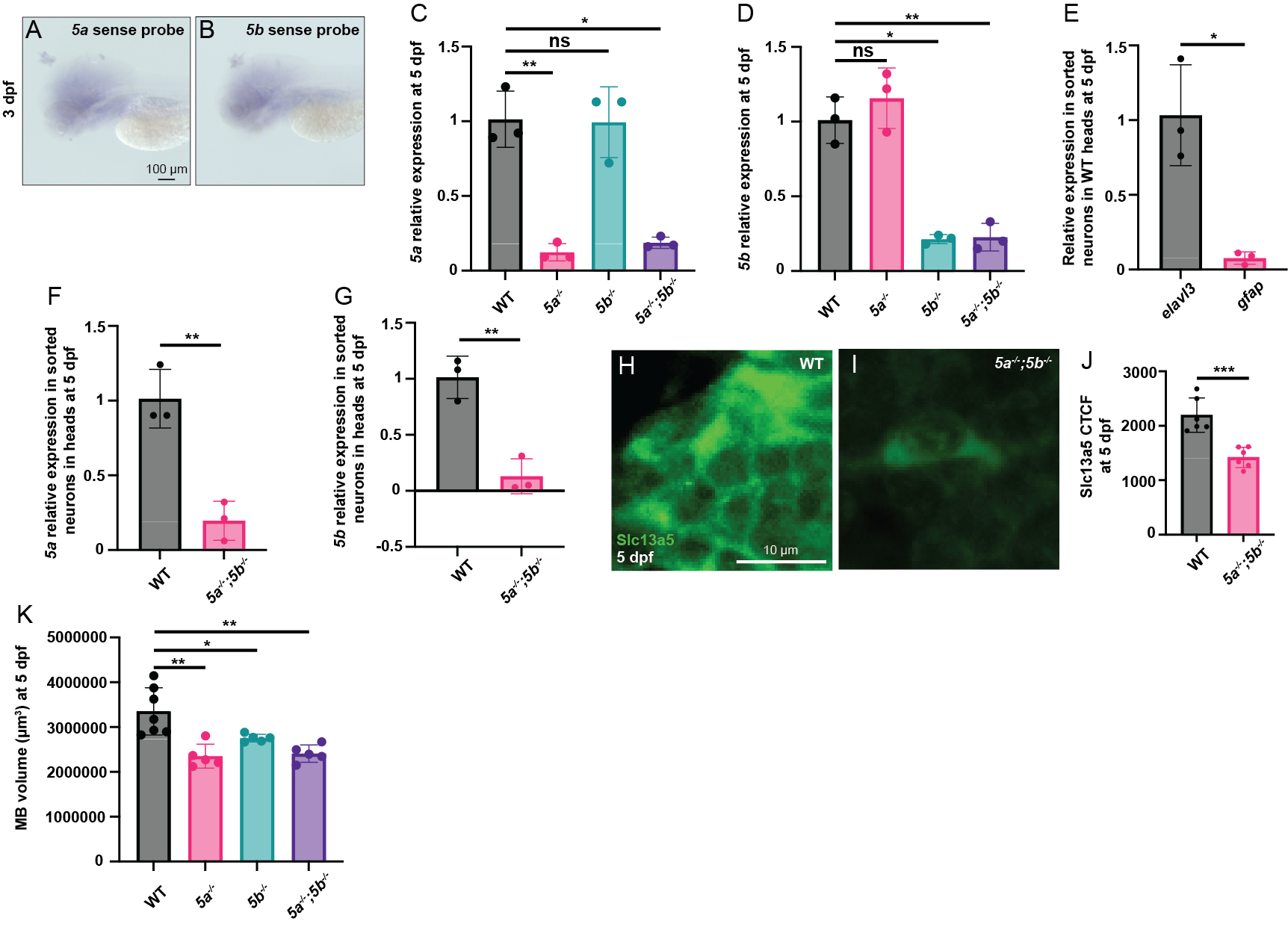

Supplement: S1 Fig — (A, B) In situ hybridization for 5a and 5b expression using sense RNA (control) probes. Negligible expression of 5a and 5b indicates that control probes used are reliable. n = 3 for each probe. (C) qPCR analysis for relative 5a mRNA expression in 5 dpf 5a−/−, 5b−/− and 5a−/−;5b−/− larvae compared to WTs. 5a is downregulated in 5a−/− and 5a−/−;5b−/− larvae compared to WTs, indicating active mRNA degradation of 5a−/− transcripts. Unchanged 5a transcript levels in 5b−/− larvae points to the absence of genetic compensation by paralog upregulation. WT, 5a−/−, 5b−/− and 5a−/−;5b−/−, n = 3 × 10 larvae pooled. (D) qPCR analysis for relative 5b mRNA expression in 5 dpf 5a−/−, 5b−/− and 5a−/−;5b−/− compared to WTs. 5b is downregulated in 5b−/− and 5a−/−;5b−/− larvae compared to WTs, indicating active mRNA degradation of 5b−/− transcripts. Unchanged 5b transcript levels in 5a−/− larvae points to the absence of genetic compensation by paralog upregulation. WT, 5a−/−, 5b−/− and 5a−/−;5b−/−, n = 3 × 10 larvae pooled. (E) qPCR analysis for relative mRNA expression of elavl3 and gfap in FACS-sorted neurons obtained from the heads of 5 dpf WTs (Tg(elavl3:ubci-Cer-sv40)). elavl3 is upregulated compared to gfap, confirming the purity of the neuronal population. WT, n = 3 × 100 heads pooled. (F and G) qPCR analysis for relative 5a and 5b mRNA expression in FACS-sorted neurons obtained from the heads of 5 dpf larvae (Tg(elavl3:ubci-Cer-sv40)). 5a and 5b transcript levels are significantly reduced in 5a−/−;5b−/− larvae compared to WTs, further validating that both the paralogs are highly expressed in the neurons compared to astrocytes. WT and 5a−/−;5b−/−, n = 3 × 100 heads pooled. (H-J) 5 dpf WT and 5a−/−;5b−/− larvae; α-Slc13a5 (green). Quantification of the fluorescence intensity (CTCF) of Slc13a5 in the brain. 5a−/−;5b−/− larval brains show a significant reduction in the expression of Slc13a5 compared to WTs. WT, n = 6; 5a−/−;5b−/−, n = 6. (K) MB volume quantification at 5 dpf; α-Slc1 [file pbio.3002499.s001.tif]

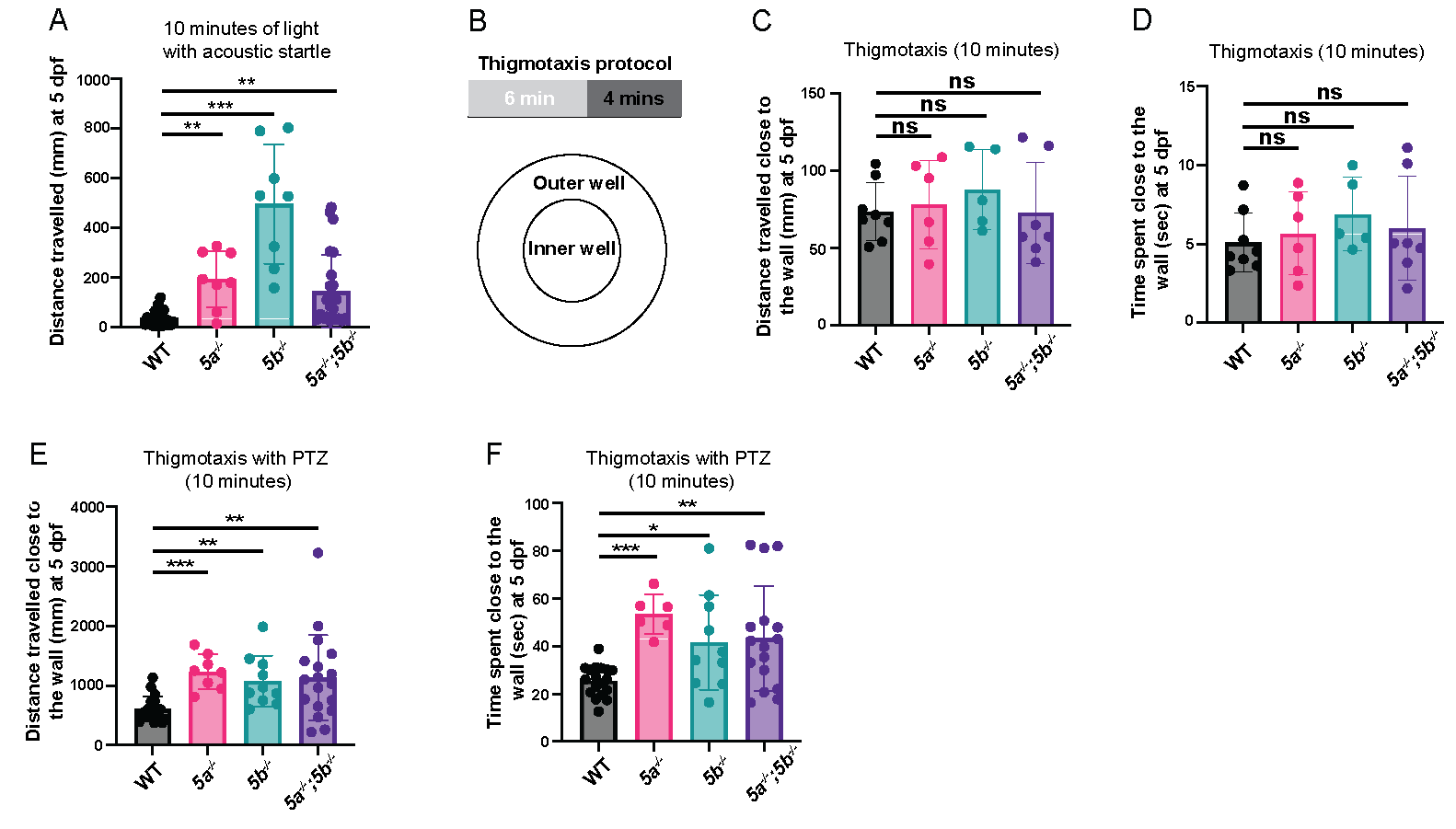

Supplement: S2 Fig — (A) Quantification of distance traveled during complete acoustic startle protocol for 10 min at 5 dpf. slc13a5 mutants move higher distances compared to WTs. WT, n = 21; 5a−/−, n = 8; 5b−/−, n = 8; 5a−/−;5b−/−, n = 24. (B) Schematic representation of thigmotaxis protocol. (C) Quantification of distance traveled close to the wall in 10 min. No significant changes were observed in the distance traveled between slc13a5 mutants and WTs. WT, n = 8; 5a−/−, n = 6; 5b−/−, n = 5; 5a−/−;5b−/−, n = 7. (D) Quantification of time spent close to the wall in 10 min. No significant changes were observed in the time spent between slc13a5 mutants and WTs. WT, n = 8; 5a−/−, n = 6; 5b−/−, n = 5; 5a−/−;5b−/−, n = 7. (E) Quantification of distance traveled close to the wall in 10 min post PTZ exposure. slc13a5 mutants swam significantly higher distance hugging the wall compared to WT. WT, n = 18; 5a−/−, n = 8; 5b−/−, n = 10; 5a−/−;5b−/−, n = 18. (F) Quantification of time spent close to the wall in 10 min post PTZ exposure. slc13a5 mutants spent significantly more time hugging the wall compared to WT. WT, n = 18; 5a−/−, n = 6; 5b−/−, n = 10; 5a−/−;5b−/−, n = 16. Data are Mean ± S.D., ns: no significant changes observed, * P ≤ 0.05, **P ≤ 0.01, ***P ≤ 0.001- Unpaired t test. PTZ, pentylenetetrazol. The data underlying this figure can be found in S1 Data. (TIF) [file pbio.3002499.s002.tif]

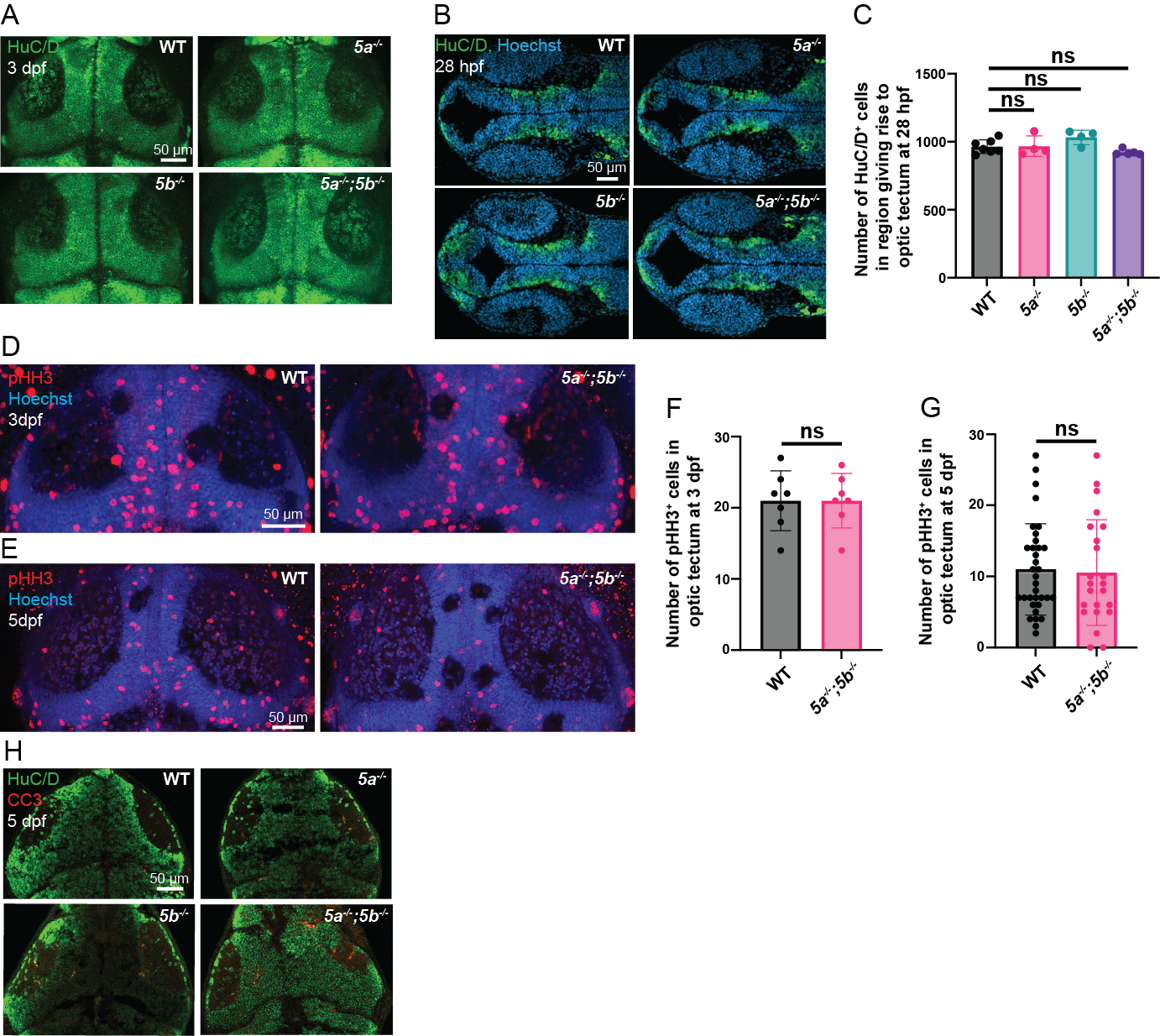

Supplement: S3 Fig — (A) 3 dpf WTs and slc13a5 mutants; α-HuC/D (green). (B) 28 hpf WTs and slc13a5 mutants; α-HuC/D (green), Hoechst (blue). (C) Quantification of HuC/D + cells in the region that gives rise to optic tectum at 28 hpf. HuC/D + cell numbers are unaffected in the slc13a5 mutants compared to WTs. WT, n = 7; 5a−/−, n = 4; 5b−/−, n = 4; 5a−/−;5b−/−, n = 5. (D-G) 3 dpf and 5 dpf WT and 5a−/−;5b−/− larvae; α-pHH3 (red), Hoechst (blue). Quantification of pHH3 + cells in the optic tectum. Cell proliferation is unchanged in the 5a−/−;5b−/− larvae compared to WTs. WT, n = 7; 5a−/−;5b−/−, n = 7 at 3 dpf. WT, n = 34; 5a−/−;5b−/−, n = 23 at 5 dpf. (H) 5 dpf WTs and slc13a5 mutants; α-HuC/D (green), CC3 (red). Data are Mean ± S.D., ns: no significant changes observed- Unpaired t test. pHH3, Phosphohistone H3. The data underlying this figure can be found in S1 Data. (TIF) [file pbio.3002499.s003.tif]

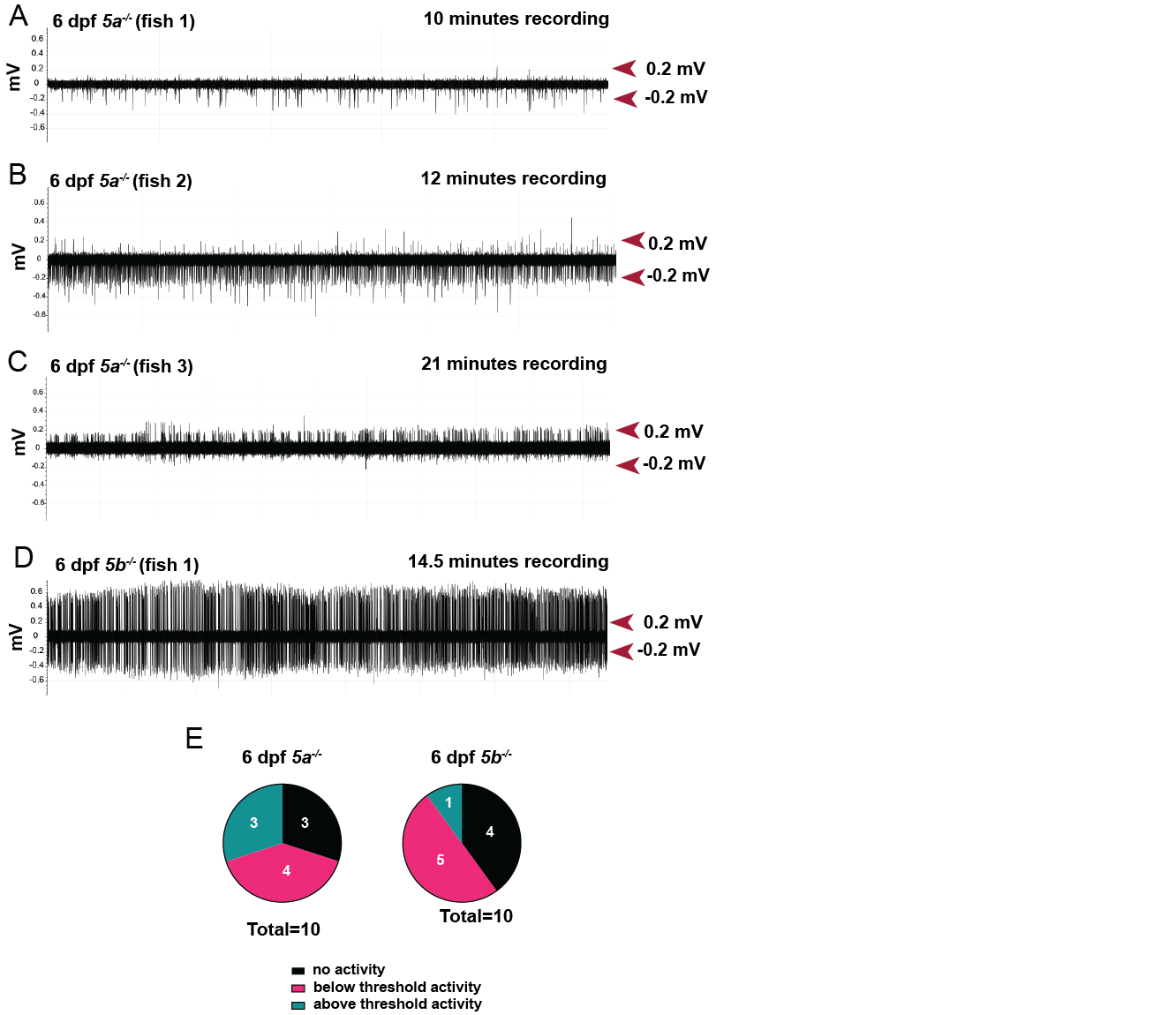

Supplement: S4 Fig — 5a−/− and 5b−/− larvae. (A-E) Representative extracellular recordings obtained from optic tectum of 6 dpf 5a−/− and 5b−/− larvae, and pie charts of proportion of 5a−/− and 5b−/− larvae showing different patterns of activity. The repetitive inter-ictal like discharges (<1s duration) with above threshold (>0.2mV), high-frequency, large-amplitude spikes seen in slc13a5 mutants are indicative of increased network hyperexcitability. slc13a5−/− larvae also displayed below threshold (<0.2mV) brain activity. (TIF) [file pbio.3002499.s004.tif]

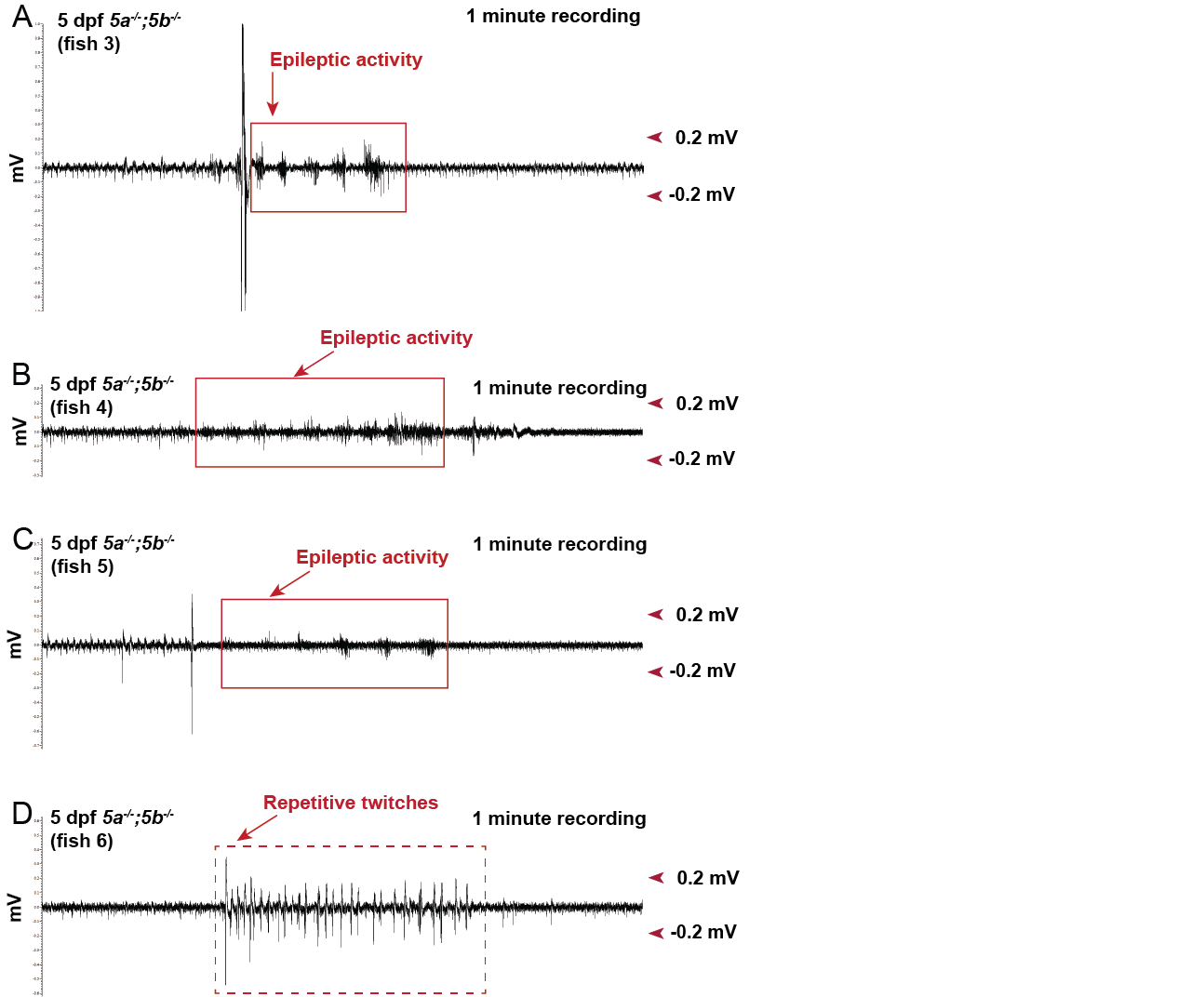

Supplement: S5 Fig — (A-D) Representative extracellular recordings obtained using NeuroProbe from optic tectum of 5 dpf 5a−/−;5b−/− larvae, showing epileptic activity, consisting of a series of short bursts, with a total duration of> 10s and repetitive twitches (>5s) at consistent frequency (~1.8 Hz) as another indication of seizures. (TIF) [file pbio.3002499.s005.tif]

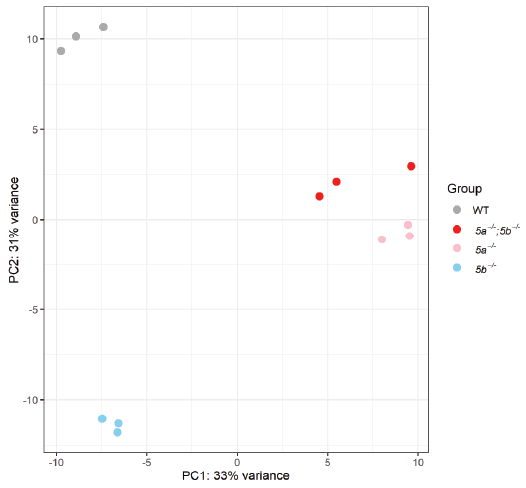

Supplement: S6 Fig — PCA plot showing that 5a−/− and 5a−/−;5b−/− are closer to each other compared to WTs and 5b−/− at transcriptomics level. PCA, Principal component analysis. (TIF) [file pbio.3002499.s006.tif]

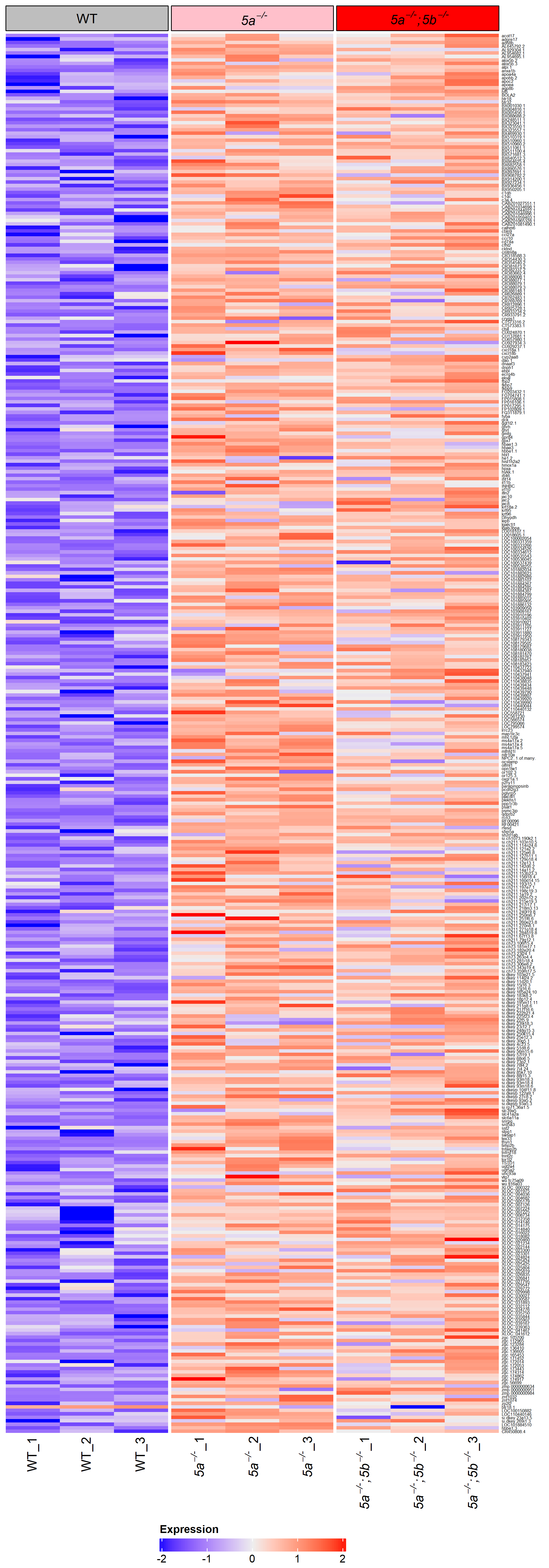

Supplement: S7 Fig — Heatmap showing a list of 400 genes significantly upregulated (padj < 0.05 and log2FC > 1.5) in 5a−/− and 5a−/−;5b−/− larvae compared to WTs. The data underlying this figure can be found in S2 Data. (TIFF) [file pbio.3002499.s007.tiff]

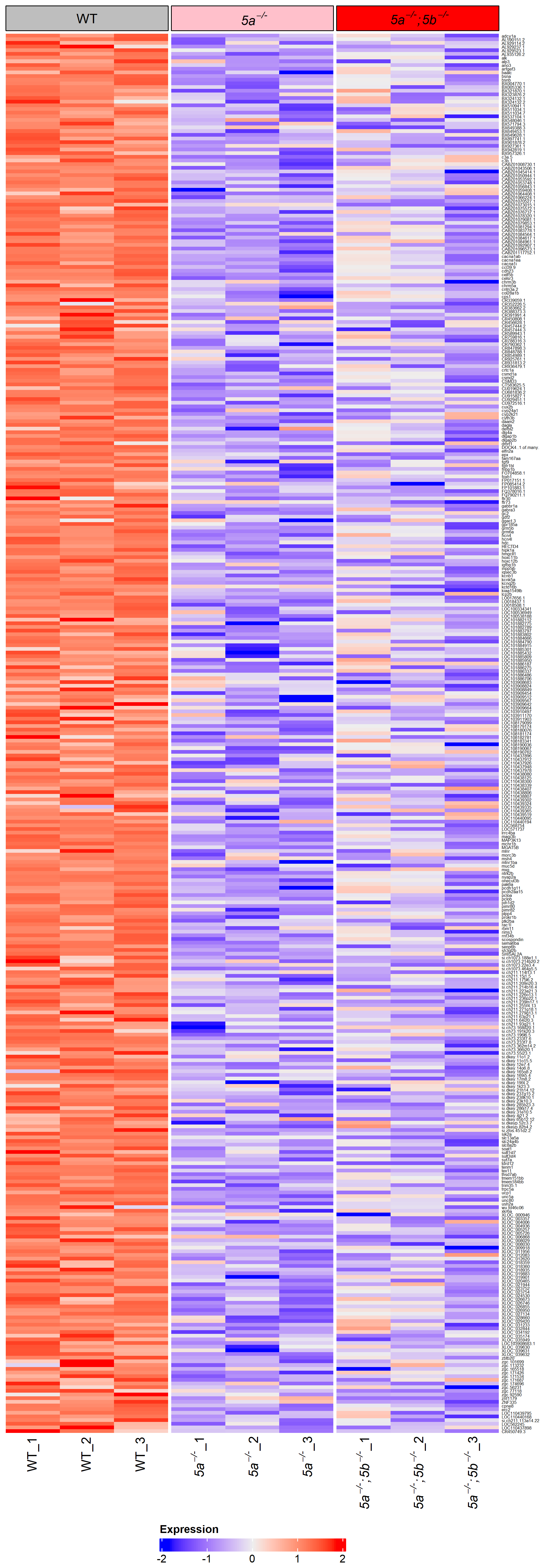

Supplement: S8 Fig — Heatmap showing a list of 380 genes significantly downregulated (padj < 0.05 and log2FC < −1.5) in 5a−/− and 5a−/−;5b−/− larvae compared to WTs. The data underlying this figure can be found in S2 Data. (TIFF) [file pbio.3002499.s008.tiff]

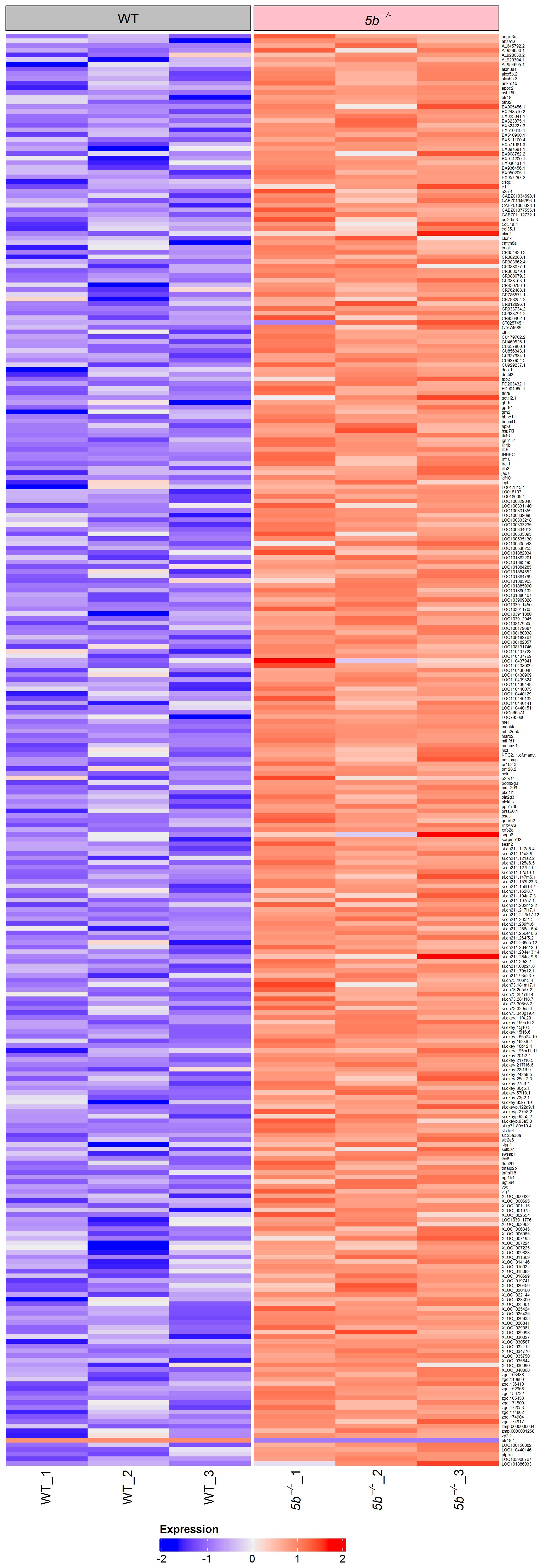

Supplement: S9 Fig — Heatmap showing a list of 304 genes significantly upregulated (padj < 0.05 and log2FC > 1.5) in 5b−/− larvae compared to WTs. The data underlying this figure can be found in S2 Data. (TIFF) [file pbio.3002499.s009.tiff]

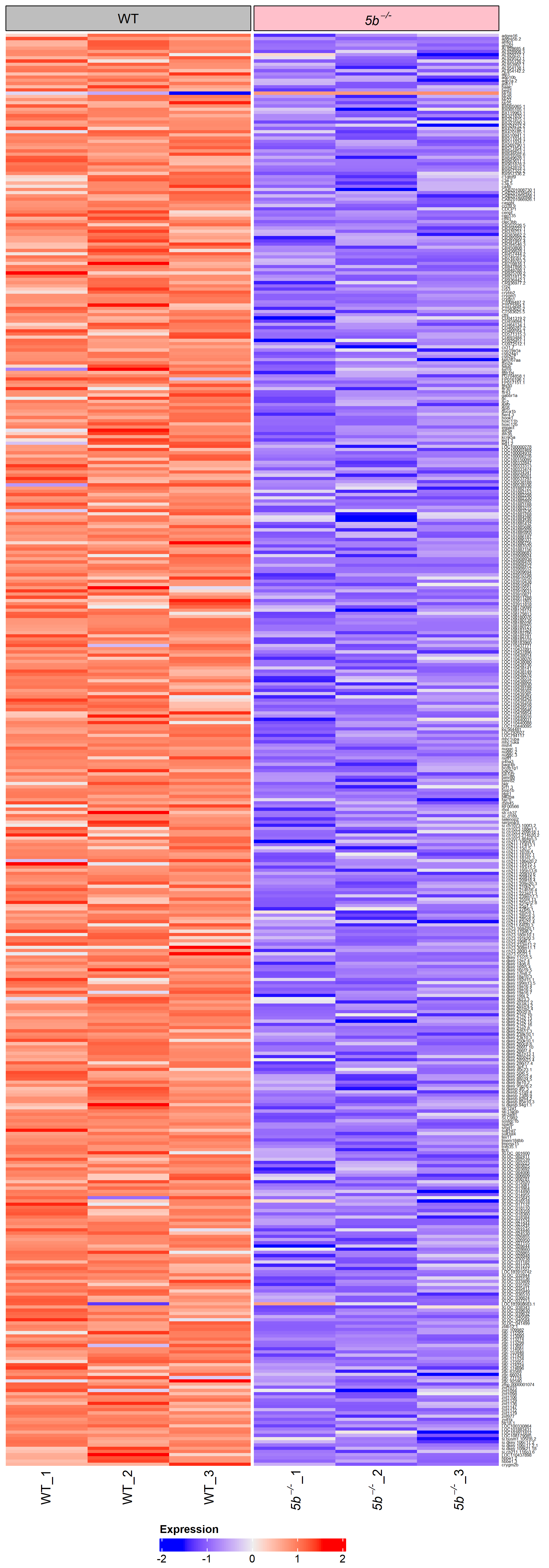

Supplement: S10 Fig — Heatmap showing a list of 444 genes significantly downregulated (padj < 0.05 and log2FC < −1.5) in 5b−/− larvae compared to WTs. The data underlying this figure can be found in S2 Data. (TIFF) [file pbio.3002499.s010.tiff]

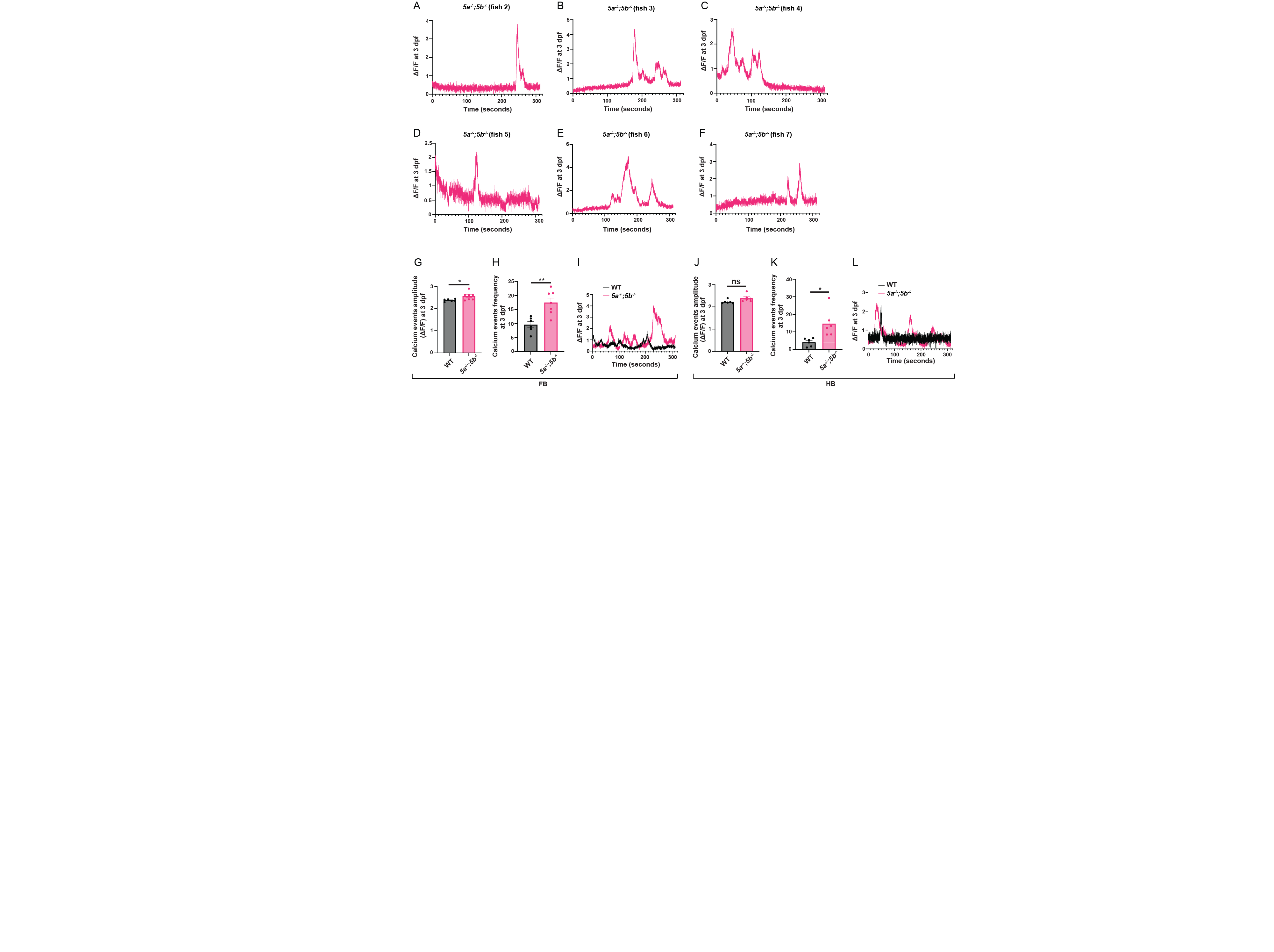

Supplement: S11 Fig — (A-F) Representative single neuron calcium traces illustrating that 5a−/−;5b−/− larvae exhibit above-threshold events in the MB. (G-I) Quantification of the amplitude and frequency of calcium events (ΔF/F > 2) and representative single neuron calcium traces at 3 dpf in the FB. 5a−/−;5b−/− larvae show significant increase in calcium events compared to WTs. WT, n = 6; 5a−/−;5b−/−, n = 7. (J-L) Quantification of the amplitude and frequency of calcium events (ΔF/F > 2) and representative single neuron calcium traces at 3 dpf in the HB. 5a−/−;5b−/− larvae show significant increase in the frequency of calcium events compared to WTs and the amplitude of calcium events remains unchanged. WT, n = 6; 5a−/−;5b−/−, n = 6. Data are Mean ± S.E.M., ns: no significant changes observed, * P ≤ 0.05, **P ≤ 0.01- Unpaired t test. The data underlying this figure can be found in S1 Data. (TIF) [file pbio.3002499.s011.tif]

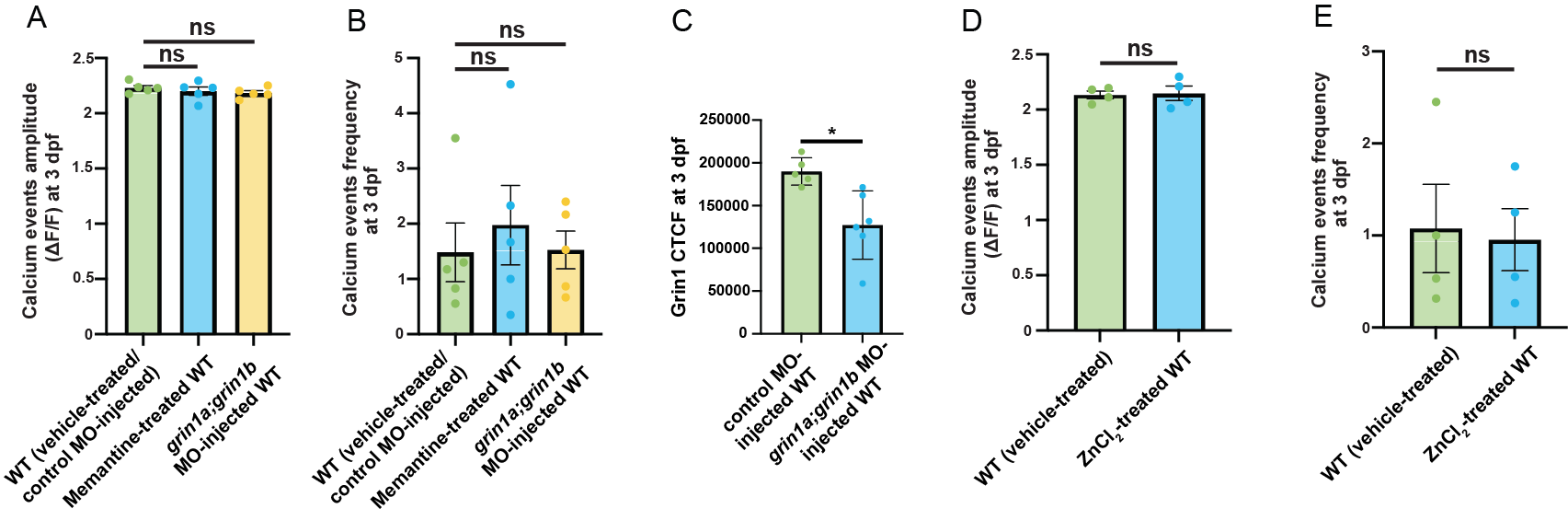

Supplement: S12 Fig — (A-B) Quantification of the amplitude and frequency of calcium events (ΔF/F > 2) at 3 dpf in the MB. Memantine treatment and grin1a;grin1b MO injections have no significant effect on the calcium events in WTs. Vehicle-treated/control MO-injected WT, n = 5; memantine-treated WT, n = 5; grin1a;grin1b MO injected WT, n = 5. (C) Quantification of the fluorescence intensity (CTCF) of Grin1 (α-Grin1). grin1a;grin1b MO injections lead to significant reduction in the expression of Grin1 compared to control MO-injected WTs at 3 dpf. control MO-injected WT, n = 5; grin1a;grin1b MO-injected WT, n = 6. (D-E) Quantification of the amplitude and frequency of calcium events (ΔF/F > 2) at 3 dpf in the MB. ZnCl2 treatment has no significant effect on the calcium events in WTs. Vehicle-treated WT, n = 4; ZnCl2-treated WT, n = 4. Data are Mean ± S.E.M. and Mean ± S.D., ns: no significant changes observed, * P ≤ 0.05- Unpaired t test. The data underlying this figure can be found in S1 Data. (TIF) [file pbio.3002499.s012.tif]

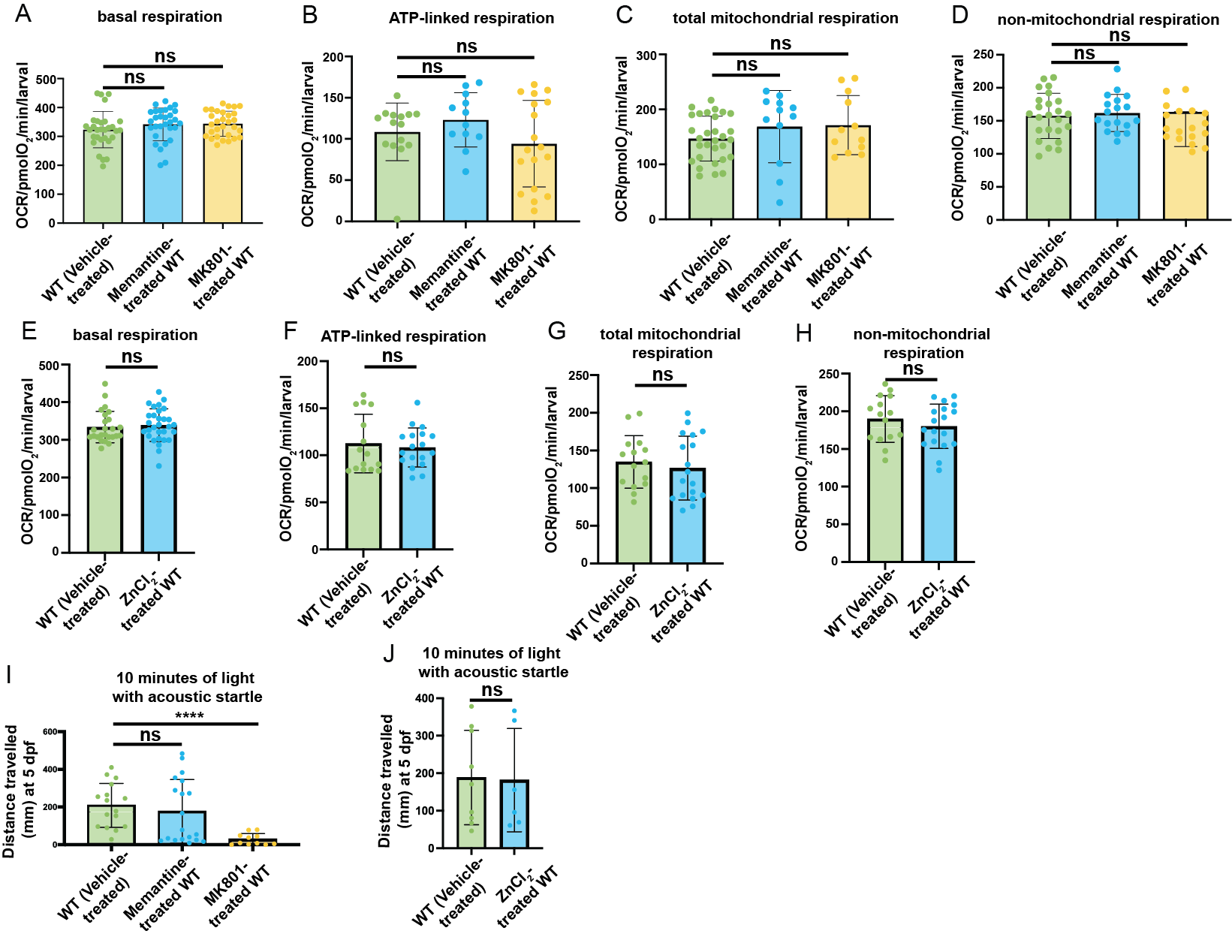

Supplement: S13 Fig — (A) Quantification of basal respiration at 6 dpf. Memantine and MK801 treatments do not affect basal respiration in WTs. Vehicle-treated WT, n = 6; memantine-treated WTs, n = 6; MK801-treated WTs, n = 6 (individual values plotted from five cycles). (B) Quantification of ATP-linked respiration at 6 dpf. Memantine and MK801 treatments do not affect ATP-linked respiration in WTs. Vehicle-treated WT, n = 5; memantine-treated WTs, n = 4; MK801-treated WTs, n = 6 (individual values plotted from three cycles). (C) Quantification of total mitochondrial respiration at 6 dpf. Memantine and MK801 treatments do not affect total mitochondrial respiration in WTs. Vehicle-treated WT, n = 10; memantine-treated WTs, n = 4; MK801-treated WTs, n = 4 (individual values plotted from three cycles). (D) Quantification of non-mitochondrial respiration at 6 dpf. Memantine and MK801 treatments do not affect non-mitochondrial respiration in WTs. Vehicle-treated WT, n = 8; memantine-treated WTs, n = 6; MK801-treated WTs, n = 6 (individual values plotted from three cycles). (E) Quantification of basal respiration at 6 dpf. ZnCl2 treatment does not affect basal respiration in WTs. Vehicle-treated WT, n = 5; ZnCl2-treated WTs, n = 6 (individual values plotted from five cycles). (F) Quantification of ATP-linked respiration at 6 dpf. ZnCl2 treatment does not affect ATP-linked respiration in WTs. Vehicle-treated WT, n = 5; ZnCl2-treated WTs, n = 6 (individual values plotted from three cycles). (G) Quantification of total mitochondrial respiration at 6 dpf. ZnCl2 treatment does not affect total mitochondrial respiration in WTs. Vehicle-treated WT, n = 5; ZnCl2-treated WTs, n = 6 (individual values plotted from three cycles). (H) Quantification of non-mitochondrial respiration at 6 dpf. ZnCl2 treatment does not affect non-mitochondrial respiration in WTs. Vehicle-treated WT, n = 5; ZnCl2-treated WTs, n = 6 (individual values plotted from three cycles). (I) Quantification of total distance traveled in [file pbio.3002499.s013.tif]
