# Supplementary material for: Modulation of NMDA receptor signaling and zinc chelation prevent seizure-like events in a zebrafish model of SLC13A5 epilepsy
Source: PLoS Biol. 2025 Apr 10;23(4):e3002499. doi: 10.1371/journal.pbio.3002499 (PMC12047791; doi:10.1371/journal.pbio.3002499)
Supplement: Attachment — Submitted filename: Dogra et al_Response to Reviewers.pdf [file pbio.3002499.s033.pdf]

UNIVERSITY OF CALGARY

CUMMING SCHOOL OF MEDICINE

Department of Medical Genetics
Alberta Children's Hospital Research Institute
Hotchkiss Brain Institute
Room 2215/Health Science Centre
Foothills Campus, University of Calgary
3330 Hospital Drive NW
Calgary, Alberta Canada T2N 4N1

T +1.403.210.6713
F +1.403.210.8757
E kurrasch@ucalgary.ca

October 14, 2024

RE: Re-submission of Research Article "PBIOLOGY-D-24-00025R1" to *PLOS Biology*

Dear Dr Nonia Pariente,

We are pleased to submit the revised version of our manuscript "Modulation of NMDA receptor signaling and zinc chelation prevent seizure-like events in *slc13a5* loss-of-function zebrafish" by Deepika Dogra et al to *PLOS Biology*. We thank the Academic Editor and all the Reviewers for the careful consideration and helpful comments.

We have addressed the reviewers' concerns either through more experiments, revision of the text, and/or our response herein. Specifically, new experimental data is now included in Fig. 1-3, 5-8, S1, S3-13 and text (highlighted in track changes version), with some sentences deleted (highlighted in track changes version) for the purpose of staying within the word limits. Please find below our responses (italicized) to each reviewer comment.

Below is a point-by-point response to each Reviewer's comment.

REVIEWER'S COMMENTS AND AUTHOR'S RESPONSES:

Reviewer: 1
This manuscript from Dogra et al. reports the phenotypic characterization of a zebrafish model of slc13a5 inactivation. The study is well constructed, and it targets an important area of biomedical research. Murine models of Slc13a5 inactivation do not provide significant translational information. As such, the introduction of alternative models is welcome.
The Authors provide a very nice description of the phenotype with behavior, cellular and metabolic assessment. They also show convincing data to link NMDA imbalance with the epilepsy-related phenotype. Final the study highlights a divergence between the epilepsy- and developmental-linked events.

We are pleased the Reviewer 1 found our paper "well-constructed", providing a "very nice description of the phenotype" and "convincing data".

UNIVERSITY OF CALGARY

Reviewer: 2
Emre Yaksi (note, reviewer 2 has signed this review) : In this study from the Kurrasch laboratory, the authors characterize zebrafish mutants for slc13a5, a citrate transporter. The authors demonstrate that the animals exhibit hyperactive behaviors, and physiological data supports increased neural activity and decreased metabolic rate. The authors also reveal increased PERK in the forebrain and present evidence for elevated calcium activity, although the location and nature of calcium signals are less clear. In the final section, the authors employ morpholinos (MO) against glun1 to illustrate that targeting NMDA receptors can rescue some of the neural excitability, behavioral, and metabolic phenotypes.
I believe that this study provides a comprehensive characterization of the mutant line, showcasing hyperactive behaviors, increased neural activity, and reduced metabolism. However, based on the presented data, I am unsure whether we are observing a seizure/epilepsy model or a hyperactivity phenotype. While animals with such high levels of activity might eventually experience seizures, the data at this developmental stage does not resemble a typical seizure phenotype, as expected from other zebrafish and epilepsy models. In my view, this study is interesting for understanding the role of citrate transporters in brain function. However, I think the authors need to better characterize their animals. If the authors claim that these animals truly experience seizures, they should demonstrate this seizure phenotype with more robust physiological and calcium experiments.
I am optimistic about the value of this study, and I truly hope some of my comments will assist the authors in improving their research.

We are pleased Dr Yaksi found our paper to be a "comprehensive characterization" and was 'optimistic about the value of this study". Yet, concerns were raised whether our slc13a5 *mutant zebrafish displayed a true seizure/epilepsy phenotype or instead hyperactivity. Thus, additional characterization was requested to demonstrate whether the* slc13a5 *mutant zebrafish "truly experience seizures", which we address with new data in Fig. 3 and S5, as well as throughout the text and in our response below. Specifically*

MAJOR COMMENTS:

1. One major concern is the authors' claim that these mutants are an epilepsy/seizure model. Clear evidence of seizures in these animals is missing. Performing calcium imaging and analyzing the data to meet standards of the field should clarify this. The examples in Figure 3D appear to be interictal activity, but have these mutant animals ever shown large ictal bursts followed by long depression periods? I could not find any evidence of such seizure-like ictal activity in the electrical or calcium recordings from mutant animals. Therefore, I am convinced that these animals are hyperactive, both behavioral, neural and metabolic recordings points towards this, but I did not find what evidence is presented for observed seizures in these mutant

UNIVERSITY OF CALGARY

zebrafish. Please see these papers, how such large ictal bursts may look during ictal bursts of calcium and electrical recordings: https://doi.org/10.1038/s41467-019-11739-z , https://doi.org/10.1002/glia.24106 , https://doi.org/10.1111/epi.17380

Thank you for this valuable suggestion. We have further investigated the electrophysiological phenotype of our mutants, and we now include new data for electrical field potentials, using a more sensitive 16-channel NeuroProbe. We show that slc13a5 *mutants exhibit a distinct epileptic pattern, consisting of a series of short bursts, with a total duration of >10s (Fig. 3K, Fig. S5A-C), and repetitive twitches (>5s) at consistent frequency (~1.8 Hz) (Fig. 3L, Fig. S5D), another indication of epileptic seizures, based on previously published work (Zdebik et al., PLOS One, 2013). These data support our conclusion that* slc13a5 *zebrafish mutants are a promising model of epilepsy. We have included all the information relevant to these new data on pages 9, 16 and 20.*

2. I would like to see the electrophysiological and calcium data from individual animals presented more effectively. As of now, we are asked to rely on the authors' classification and a few example traces, but it is crucial to present data from individual animals, at least as a supplemental figure. This would allow a closer examination of the potential causes of such large-scale variability across animals. I would also appreciate an explanation why many mutants do not exhibit physiological phenotypes, while mutant behavior seems to show a big differences from controls.

We appreciate this comment and here we show expanded electrophysiological (Fig. 3G-I, Fig. S4, Fig. 3K-M, Fig. S5) and calcium data (Fig. 6A-C, Fig. S11A-F) from individual slc13a5 *mutants. As for the variability, we speculate that the incomplete penetrance of the electrophysiological phenotypes in the mutants is because we recorded in larval zebrafish and seizure-like events likely increase in frequency as the animals age. In* Scn1a$^{+/-}$ *Dravet mice, for example, it is common for seizures to increase in severity around the time of weaning (reviewed in Griffin et al., Frontiers in Pharmacology, 2018) and we propose a similar pattern in these* slc13a5 *zebrafish mutants. In support, our survival curve data shows that only 5-15% of the mutants are alive by adulthood despite appearing active at the larval stages, perhaps consistent with an increasing epilepsy phenotype. We now include these new data and explanation on pages 9, 11 and 16.*

3. The authors make an argument towards unchanged neurogenesis but increased cell death. I am fine but in that case the authors needs to make more a solid quantification of neurogenesis. For this the authors can use Phosphorylated Histone H3 staining. effective anti-pH3 antibody staining can be found here: https://doi.org/10.1101/2024.02.01.578354 or here https://doi.org/10.1126/sciadv.aaz3173 . PH3 staining will label diving cells, and I would

UNIVERSITY OF CALGARY

recommend to do this staining at few different stages between 2-5 days.

Thank you for this suggestion. pHH3 immunostainings further show that cell proliferation at primary (3 dpf) and secondary (5 dpf) neurogenesis is not increased in slc13a5 *mutants (Fig. S3D-G). All the information relevant to these new data are mentioned on pages 8 and 21.*

4. The increase in amplitude and frequency of calcium events in mutants is interesting, but it is not clear how these events are detected, based on what criteria, and how robust and comparable these are across animals and conditions. More data presentation of such detected events over calcium traces would be helpful. Defining event thresholds in unbiased ways and performing complementary analyses would provide more confidence in the robustness of these findings.

We appreciate this comment and now provide a more detailed explanation on pages 22 and 23. Briefly, we noticed that at 3 dpf, most of our WTs stayed within the amplitude (ΔF/F) of ≤ 2, while our slc13a5 *mutants showed an amplitude of ≥ 2. Therefore, we considered a threshold amplitude value of 2, i.e., anything > 2 was considered as "calcium event". Notably, 2 is a higher threshold (and thus, more rigorous) value than other previous studies (Liu and Baraban, eNeuro, 2019; Brenet et al., Cells, 2024). We calculated the average amplitude and average frequency of calcium events of all the neurons captured by selecting regions of interest (ROI) in Fiji for each zebrafish, focusing on different brain regions (forebrain, midbrain and hindbrain).*

5. The brain regions analyzed for calcium signals (presumably the optic tectum) need clarification. Are these signals, or at least conclusions of the results, similar in other brain regions such as the forebrain and brainstem? The authors showed a significant increase in pERK signaling in the forebrain but not in the midbrain and hindbrain of mutants. It is unclear if these regions with the largest increase in neural activity also show the largest PERK signals and the largest amount of neural death. This link is not clearly shown or explained.

Initially we analyzed calcium events only in the midbrain (Fig. 6A-C) even though the pERK data showed elevated pERK activity across all three brain regions -- the forebrain, midbrain and hindbrain – in the slc13a5 *mutants (as indicated by green signal in Fig. 6D). Here, we add new data showing calcium events in 3 dpf WTs and* slc13a5 *mutants in the forebrain and hindbrain. Consistently, we observed a significant increase in the amplitude and frequency of calcium events in the forebrain and a significant increase in the frequency of calcium events in the hindbrain (Fig. S11G-L). In the main text, we focus on the midbrain region given the higher expression of the* slc13a5 *paralogs in this brain region and reduction of the mutant midbrain size at later developmental stages (Fig. 1A-F, Fig. 1T-W, Fig. S1K). These new data are discussed on pages 11 and 22.*

6. It is not clear to me how hyperactive mutant animals with more robust swim behaviors exhibit lower levels of metabolic and basal respiration signals. The mechanisms explaining this contradiction need clarification.

Although perhaps counter-intuitive, bioenergetics levels does not directly correlate with neuronal activity. That is, lower bioenergetics can manifest as hyperexcitability and higher bioenergetics can result in neuronal dampening. What is important is that mitochondrial output is altered from baseline, thereby disrupting normal neuronal homeostasis that manifests differently presumably due to the neuronal fates most affected.

Indeed, our previous work studying kcna1$^{-/-}$ *genetic mutants and* kcna1 *morphants illustrates this pattern. The* kcna1 *mutants show a significant reduction in various neurometabolic parameters and concomitant neuronal hyperexcitability (Dogra et al., Epilepsia, 2023). Conversely, the kcna1a morpholino-injected zebrafish display an increase in bioenergetic readouts, including basal respiration, ATP-linked respiration and total mitochondrial respiration and neuronal hyperexcitability (Ibhazehiebo et al., Brain, 2018). We propose these opposite bioenergetic phenotypes arise due to an acute (e.g.,* kcna1a *morphants) or persistent (*kcna1a *loss-of-function mutants) effect on neuronal homeostasis. Such differences in patterns of metabolic deficits have also been reported previously using other zebrafish models of epilepsy (Banerji et al., Brain Communications, 2021). Ultimately, regardless of whether the bioenergetics outputs are increased or decreased, mitochondrial function appears to be compromised across most epilepsy models.*

7. The MO experiments in Figure 4E-H suggest a role of these proteins in neural excitability. However, presume the role of Glun1 in neural excitbity is well known. So it is not clear what we are learning from these experiments and how it relates to the remaining part of this study?

We agree with this comment and here we show Grin1 expression relative to Slc13a5 and that suppressing Grin1 signaling rescues various slc13a5 *mutant phenotypes, consistent with an interaction between NMDA receptor (Grin1) signaling and* slc13a5 *mutations (pages 12 and 16). Specifically we now show:*

a) Slc13a5/Grin1 co-localization in the midbrain of WT zebrafish (Fig. 6E-G)
b) Grin1 expression is increased in the neurons in midbrain of slc13a5 *mutants compared to WTs (Fig. 6H-K)*
c) NMDA antagonist (memantine) treatment and grin1a;grin1b *morpholino injections rescue dysregulated calcium activity (Fig. 7A-C), disrupted bioenergetics profile (Fig. 7G-J) and impaired startle response (Fig. 7O) in* slc13a5 *mutants*

UNIVERSITY OF CALGARY

8. What experiments demonstrate a role for NMDA receptors in slc13a5 mutant dysregulation of Ca+2 signalling ? I guess for making this statement one needs to do transcriptome or proteome analysis, isn't it ?

As discussed in the previous comment, we show that NMDA antagonist (memantine) treatment and grin1a;grin1b *morpholino injections rescue dysregulated calcium activity (Fig. 7A-C) in* slc13a5 *mutants, signifying an interaction between NMDA signaling and* slc13a5 *mutant dysregulation of Ca+2 events.*

Further, we conducted bulk RNA sequencing on 5 dpf larval heads, and show that genes involved in calcium ion import are downregulated in our slc13a5 *mutants compared to WTs (Fig. 5B), potentially due to decreased NMDA receptor-mediated calcium influx into the cells. We did not observe changes in NMDA signaling in our dataset; however, the data was generated from pooled heads, which can mask small transcript changes in specific cell populations. We have this new transcriptomic data on pages 10 and 23.*

MINOR COMMENTS:

1. In general, slc13a5 mutants move a lot more even without acoustic stimulation. Therefore, the phenotype looks more like increased swim behavior rather than increased acoustic startle. To show increased acoustic startle, the authors should demonstrate and quantify transient increases in swim behaviors upon acoustic stimulation compared to the baseline period preceding the acoustic stimulation.

Thank you for this suggestion. We now show the behavior data during baseline periods and acoustic startle periods, separately. Our data indicate that slc13a5 *mutants exhibit increased swim behavior, both in the absence of an external stimuli as well as due to acoustic startle (Fig. 2B). We mention these new data on pages 6 and 7.*

2. For circadian behaviors, please display the time course of day/night swim cycles for the recorded animals rather than just the average swim distance during the night. Please refer to Jason Rihel's paper for an example of how to display such sleep data.

We now show the circadian behavior from 4 dpf to 6 dpf, including 10 hours of night swim cycles and 6 hours of day swim cycles (Fig. 2D) and discuss on page 7.

3. Please note the concentration of PTZ challenge in figure S2.

We have now added the PTZ concentration (5 mM) to the results section on page 7.

4. Do the results of Figure 3A/B also correlate with the general ratio/distribution of glutamatergic and GABAergic neurons in the brain?

Since our lab did not have access to transgenic zebrafish lines marking excitatory and inhibitory neurons, we labelled the glutamatergic and GABAergic neurons using immunostainings for vGlut1 and Gad67 (Fig. 1I and J), respectively. We further attempted to compare the vGlut1$^+$ and Gad67$^+$ neuron numbers between WTs and slc13a5 *mutants; however, we noticed that not all the inhibitory and excitatory neurons are marked with these antibodies. Therefore, we did not find this method reliable enough to quantify neuronal fates.*

5. The authors wrote, "We speculate that a range of electrical activities, i.e., above and below threshold spikes, observed in our mutants could be because of the stochastic nature of seizures." It is not clear what is meant by this statement and how it explains the lack of evidence for large ictal bursts followed by postictal depression.

We have now provided a better discussion of our data on pages 9 and 16.

6. The sentence "Pharmacologically, we exposed WT and mutant zebrafish larvae to meantime, an established NMDA antagonist used in other zebrafish studies" seems incomplete. Additionally, is there a figure for the drug experiment? Please name the drug in the main text.

We mention the drug "memantine" in the results section on page 12 and the details of drug incubation in the methods section on page 19.

Reviewer: 3

Quynh Anh Nguyen (note, reviewer 3 has signed this review): Dogra et al. characterize their newly created zebrafish model of SLC13A5 deficiency and show that it displays many behavioral features seen in humans with mutations of this gene. They find that their slc13a5 mutants show increased neuronal excitation, with increased glutamatergic and decreased GABAergic gene expression, manifesting in the occurrence of inter-ictal like discharges seen in a subset of their mutant animals. The authors go on to show that increased NMDA receptor function underlies the abnormal calcium activity and behavioral phenotypes observed, demonstrating rescue of these phenotypes through either pharmacological or genetic blockade of NMDA receptors. Altogether, this study is novel and innovative, providing a valuable zebrafish model to use in studying a disease mechanism for which prior mouse models had significant limitations, and identifying a possible mechanism for the etiology of epilepsy associated with SLC13A5 deficiency in humans. The following major comment and minor

UNIVERSITY OF CALGARY

comments will help provide further clarification and enhance the study for publication in PLOS Biology.

We thank Dr Nguyen for finding our study "novel and innovative" and for providing suggestions to help enhance our study for publication. Below we address specific concerns:

MAJOR COMMENT:

1) The main limitation of this model is the limited epilepsy phenotype displayed, with only a minority of mutants showing inter-ictal like discharges and no seizures observed in any of the mutants. In addition, it is unclear to what extent the calcium events imaged correlate to this abnormal electrophysiological activity (is it possible to do both calcium imaging and extracellular field recordings simultaneously?). Furthermore, the title of the study states that the epileptic phenotypes are rescued by NMDA blockade, but the authors only show rescue specifically for the calcium events, bioenergetics, and startle response and do not show whether there are less inter-ictal like discharges. Given the limitations with studying the spontaneous aspects of epilepsy in this model, notably seizures, it would be worth assaying whether the mutants show increased seizure susceptibility to PTZ exposure (do they display a more severe seizure phenotype or are more susceptible to seizures?). If they do, then this could be used to test whether NMDA blockade is able to decrease this seizure susceptibility and provide more support for their conclusion that this mechanism is responsible for the epilepsy-related phenotypes in their model.

Thank you for these valuable suggestions. To address the concern that this model displays limited epilepsy phenotype with inter-ictal discharges and no seizures, we now include new data. Please see our responses to major comments #1 and #2 for Reviewer 2 above.

Unfortunately, we are unable to assay calcium fluctuations and extracellular field recordings simultaneously as the calcium imaging camera is located on a specialized microscope, and not our electrophysiology rig. Instead, we now show new electrophysiological (Fig. 3G-I, Fig. S4, Fig. 3K-M, Fig. S5) and calcium (Fig. 6A-C, Fig. S11A-F) data from individual slc13a5 *mutants, which helps to better appreciate the strength of these phenotypes. We discuss these new data on pages 9 and 16.*

Further, we tried to collect electrophysiological rescue data with memantine treatment but were unsuccessful due to the low electrophysiological penetrance in our mutants. Nevertheless, below is a successful example (n=1), showing extracellular field potentials measured from the optic tectum of an agarose-immobilized 6 dpf $5a^{-/-}$;$5b^{-/-}$ *zebrafish. We show that at baseline, an untreated* $5a^{-/-}$;$5b^{-/-}$ *mutant exhibits repetitive inter-ictal like discharges (<1s duration) with high-*

frequency, large-amplitude, above threshold (>0.2mV) spikes (Rebuttal Fig. 1) that is blocked by the addition of 50 µM Memantine in this same zebrafish, suggesting a role for NMDA signaling in slc13a5$^{-/-}$ *hyperexcitability.*

<u>Rebuttal Figure 1</u>

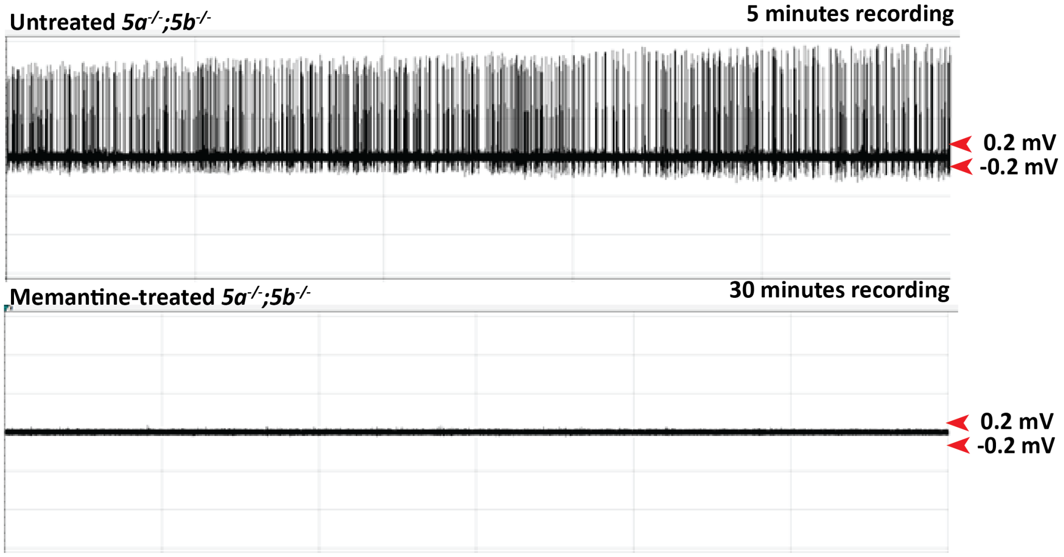

Thank you for the suggestion to use PTZ treatment to induce a stronger phenotype in the slc13a5 mutants. We include data showing that an acute treatment of 5 mM PTZ followed by exposure to thigmotaxis protocol (6 minutes light and 4 minutes darkness) leads to an increase in the distance travelled and time spent hugging to the wall in the slc13a5 *mutants (Fig. S2E and F), suggesting these mutants became more anxious. However, we did not attempt a prolonged exposure of our mutants because we were concerned that the antagonism of GABAergic inhibitory neurons with PTZ combined with the inhibition of NMDA signaling on excitatory neurons with memantine would be lethal, or at least toxic, to the zebrafish and might not lead to interpretable data.*

Overall, we demonstrate improvements in dysregulated calcium events (a proxy for neuronal hyperexcitability), disturbed neurometabolism (previously exhibited by other zebrafish epilepsy models: Ibhazehiebo et al., Brain, 2018; Banerji et al., Brain Communications, 2021; Dogra et al., Epilepsia, 2023) and impaired startle response (another feature of epileptic zebrafish as per Dogra et al., Epilepsia, 2023) that supports our hypothesis that suppressing NMDA receptor signaling rescues epileptic phenotypes in slc13a5 *mutants.*

UNIVERSITY OF CALGARY

MINOR COMMENTS:

2) The images in Fig. S1A and B were taken at 3dpf and additional images taken at 5dpf should be provided to help with comparisons.

We now include sections of 5 dpf in situ hybridization as insets in Fig. 1E and F.

3) Quantification of the cell type characterization in Fig. 1C and D should be provided.

Here, we quantified the Slc13a5^{+}/HuC/D^{+} and Slc13a5^{+}/Gfap^{+} cells in Fig. 1K and updated the text on page 5.

4) Fig. 2, panels G and H: the increase in number of HuC/D+ cells between 3 and 5dpf across all genotypes suggests that there is still neurogenesis occurring even in the mutants during this time period. Are there any markers they could stain for to check that the rate of neurogenesis is not altered between WT and mutants and could contribute to the decrease in neurons seen in the mutants?

Thank you for this suggestion. We now use pHH3 immunostainings to demonstrate that cell proliferation at later stages, including 3 dpf and 5 dpf, is not affected in slc13a5 *mutants (Fig. S3D-G). We discuss these data on pages 8 and 21.*

5) Why was calcium imaging done at 3dpf instead of at 5dpf, which would be more consistent with the timeline of their other assays?

It was necessary to conduct the calcium imaging at 3 dpf in the morpholino-based experiments because the efficacy of morpholinos is diluted at later larval stages, confounding results. Therefore, we continued with 3 dpf to keep a consistent time point for calcium imaging across all set of animals (e.g., untreated/uninjected, morpholino-injected, drug-treated, etc.). Additionally, we observed that neuronal numbers start to decline in the mutants at 3 dpf (Fig. 2G), aligning this earlier timepoint with peak neuronal functioning.

6) More n's are needed for Fig. S6A and B.

We have now added more animals to strengthen Fig. S12A and B.

7) Does knockout of only 5a expression affect 5b expression and vice versa?

Thank you for this relevant question. In this revised paper, we performed qPCRs for each paralog in the other paralog's mutant. We observed that 5a *transcript levels were unchanged in* 5b$^{-/-}$ *larvae, demonstrating a lack of genetic compensation via paralog upregulation (Fig. S1C). Similarly,* 5b *transcript levels also were unchanged in* 5a$^{-/-}$*, further consistent with a lack of genetic compensation (Fig. S1D). We mention these new data on pages 5 and 6.*

Reviewer: 4
Dogra et al. use zebrafish to model a form of infantile epileptic encephalopathy caused by mutations in the sodium-dependent citrate transporter slc13a5, with the goal of discovering disease etiology. The authors make single and double CRISPR knock-outs of paralogs slc13a5a and slc13a5b which survive the early larval period but die by 30 days of age. They describe a range of brain phenotypes: reduced brain size, reduced neuron number, increased neuronal cell death, increased behavioral excitability and motility, increased synthesis of excitatory and decreased synthesis of inhibitory neurotransmitters, increased overall neuronal activity, and generally reduced metabolic flux.

Based on their findings, the authors propose a model whereby increased extracellular citrate levels in the absence of the slc13a5 transporter chelates zinc which is normally required to regulate NMDA signaling. Consistent with this, knocking down NMDARs pharmacologically or with antisense Morpholinos rescues the slc13a5 double mutant phenotype.

The paper introduces a new animal model that recapitulates aspects of the epileptic phenotype of humans with mutations in slc13a5. However, the phenotypic characterization is superficial and the connection between the various aspects of the mutant phenotype needs to be strengthened. The robust suppression of the phenotype by NMDA block is intriguing but it is insufficient support for the zinc chelator model, since suppressing a citrate-induced increase in neuronal activity and behavior by blocking NMDA could be quite indirect. While this new animal model may be developed into a tool for further research into epileptic phenotypes, as it stands the paper fails to sufficiently elucidate the etiology of the disorder for publication in PLoS Biology.

We thank Reviewer 4 for recognizing that our paper 'introduces a new model that recapitulates aspects of the epileptic phenotype of humans with mutations in slc13a5*' and for providing a detailed review of our study. Here we address their criticisms and suggestions. In particular:*

1) the zinc chelator hypothesis is not sufficiently well supported by the finding that blocking NMDA rescues slc13a5 mutant phenotypes. A more direct test of the role of zinc as a key component of the pathway is required.

UNIVERSITY OF CALGARY

Thank you for this valuable suggestion. In this revised submission, we conduct the following experiments to provide direct evidence for a role of zinc in slc13a5 *mutations and to strengthen this zinc chelator hypothesis:*

a) We use a fluorescent zinc indicator, Palm-ZP1 (Radford et al., Chemical Science, 2013), and show that slc13a5 *mutants exhibit a reduction in zinc levels at the plasma membrane compared to WTs. Further,* $ZnCl_2$ *treatment leads to enhanced Palm-ZP1 fluorescence in the mutants (Fig. 6L-R). These data point towards possible zinc chelation in the* slc13a5 *mutants. These new data are discussed on pages 12, 16, 21 and 22.*

b) We also show that $ZnCl_2$ *treatment rescues dysregulated calcium activity (Fig. 7D-F), disturbed bioenergetics (Fig. 7K-N) and impaired startle response (Fig. 7P) in* slc13a5 *mutants, further supportive of a potential zinc chelation in* slc13a5 *mutations. These data are found on pages 13, 14, 16, 17 and 19.*

2) The stated rationale for knocking both slc13a5a and a5b down is that they may function redundantly or partly redundantly. This predicts more severe phenotypes in the double mutant than either single mutant. Regarding lethality this is observed: double mutants die sooner than either single mutant. However with respect to brain size, neuron numbers, cell death and vglut2a expression the double mutant is the same as either single mutant. Inexplicably, with respect to behavior the single slc13a5b mutant is more excitable than the double mutant. Most strangely of all, with respect to gad1b expression and electrophysiological activity, double mutants are much less severely affected than either single mutant. The authors do not note these unexpected genetic relationships and haven't done the statistics on them, but they are very obvious and need to be explained.

We appreciate this comment and to understand better their similarities and differences, we conducted bulk RNA sequencing on 5 dpf WT, 5a$^{-/-}$, 5b$^{-/-}$ and 5a$^{-/-}$;5b$^{-/-}$ *larvae. This sequencing revealed that* 5a$^{-/-}$ *and* 5a$^{-/-}$;5b$^{-/-}$*larvae are transcriptionally more similar to each other than either is to* 5b$^{-/-}$*. All three mutants are distinct than WTs (Fig. S6). This could be a possible reason for* 5a$^{-/-}$;5b$^{-/-}$ *larvae not showing more severe phenotypes compared to single* 5a$^{-/-}$*, or even* 5b$^{-/-}$ *larvae. We now discuss their genetic relationships in more detail on pages 10, 16 and 23.*

Further, in regards to the electrophysiological phenotypes, we now include new data for electrical field potentials, using a more sensitive 16-channel NeuroProbe electrode. We show that 5a$^{-/-}$;5b$^{-/-}$ *larvae exhibit a distinct epileptic pattern, consisting of a series of short bursts, with a total duration of >10s (Fig. 3K, Fig. S5A-C), and repetitive twitches (>5s) at consistent frequency (~1.8 Hz) (Fig. 3L, Fig. S5D), another indication of epileptic seizures, based on previously published work (Zdebik et al., PLOS One, 2013). We also discuss that the possible*

reason for incomplete penetrance of the physiological phenotypes in the mutants at larval stages could be perhaps due to the fact that seizures increase in the mutants more frequently as they age. Our survival curve data showing that only 5-15% mutants make it up to adulthood strongly supports this speculation (Fig. 1X). These data can be found on pages 9, 16 and 20.

Finally, we would not expect that phenotype of a single mutant is simply a reduced version of a double mutant for a few reasons:

(1) *Functional redundancy does not equal simple additivity. Although* 5a *and* 5b *may have overlapping functions, their individual contributions to a biological process is not necessarily additive, with each gene having unique, context-specific roles or thresholds of activity. Depending on the assay used, the double mutant may reveal emergent effects that are qualitatively different than from what can be predicted by a simple reduction in the activity of either gene singly*
(2) *In a single mutant, there may be buffering mechanisms at play where the paralog (e.g.,* 5a *in a* 5b *knockout) can recruit additional pathways to mitigate the loss of function. These compensatory effects can mask the full extent of the pathway disruption, leading to a phenotype that is less severe or different in nature from the double knockout*
(3) *The interactions between* 5a *and* 5b *may be non-linear, meaning that the functional relationship between them involves synergistic or antagonistic effects. This suggests that* 5a *or* 5b *act in pathways that cannot be fully understood by examining phenotypes in the single gene knockout*

Taken together, it is not surprising that the single gene mutants display phenotypes distinct from each other and from the double knockout, as we show throughout this paper.

3) The characterization of slc13a5a/b expression is superficial. Slc13a5 expression is stated to be enriched in the midbrain (Fig. 1A, S1A,B) but in fact appears to be throughout the brain; a sense control probe should be included in Fig. 1A (mysteriously, two scRNA-Seq atlases show little (a5a) or no (a5b) expression of either paralog in the brain). Later, using a polyclonal rabbit antibody raised against human Slc13a5, they show expression is shown to be quite specific in a subset of neurons and glial cells (Fig. 1C, D, Fig. 4E) but what and where these neurons are is not determined. First, the specificity of the antibody for zebrafish slc13a5 proteins should be validated by staining of single and double mutants. Then the specific expressing neurons should be identified and shown to be relevant to the other phenotypes. (Additional note: the Grin1 expression in the neurons in Fig. 4E is not detectable; separate channels should be shown).

We now include in situ hybridization data using sense probes for 5a *and* 5b*, showing negligible signal at 3 dpf (Fig. S1A and B), further validating the specificity of antisense probes conducted*

previously in Fig. 1A-F. We also show that 5a *and* 5b *transcripts are expressed in FACS-sorted neurons at 5 dpf and their expression declines in* $5a^{-/-}$;$5b^{-/-}$ *larvae compared to WTs (Fig. S1E-G). We discuss these new data on pages 5, 6, 18 and 19.*

We also used a new Slc13a5 antibody (PA5-113058, Invitrogen) showing stronger signal and have replaced the data of $Slc13a5^{+}/HuC/D^{+}$ *and* $Slc13a5^{+}/Gfap^{+}$ *co-expression. We also now show that a higher percentage of neurons express Slc13a5 compared to astrocytes (Fig. 1G, H and K). We further demonstrate that both excitatory and inhibitory neurons express Slc13a5 (Fig. 1I and J). Notably, the expression of Slc13a5 in both excitatory and inhibitory neuronal populations points to the qPCR-based differences in vGlut2a and Gad1b transcript levels in Fig. 3A and B. We validate Slc13a5 antibody specificity by showing reduced Slc13a5 fluorescence in* $5a^{-/-}$;$5b^{-/-}$ *larval brain compared to WTs (Fig. S1H-J). These data were all obtained from the midbrain region and can be found on pages 5, 6 and 21.*

Altogether, our new data provides a better characterization of 5a *and* 5b *expression in the brain. Further, as suggested by the reviewer, we now include images from separate channels to show Slc13a5/Grin1 co-expression in Fig. 6E-G.*

4) The description of mutant phenotypes is disjointed and superficial. Which cells in the midbrain are dying and missing? Are they the same neurons (or astrocytes?) that express slc13a5? Which neurons show increased GCaMP signals? Are they the same as the slc13a5-expressing neurons? What aspect of midbrain size is reduced? The measurement is of an (unstated) linear dimension whereas a volume or at least an area would more fully capture the size of the regions. The metabolic analysis is also very superficial, being of whole fish, while the metabolic abnormalities of the epileptic brain are specifically in the brain. Some correlate of metabolic defects in the brain, and specifically in and around the slc13a5-expressing neurons should be included. Finally, the model is that increased excitability is the direct consequence of excess extracellular citrate causing increased NMDA signaling. Yet the authors also describe a strong increase in glutamate synthesis (vgut2 expression). What is the connection between the two excitatory phenotypes?

We show an increase in cellular apoptosis (acridine $orange^{+}$ *cells) as well as increased neuronal cell death (percentage* $CC3^{+}/HuC/D^{+}$ *cells) in the optic tectum (midbrain) of* slc13a5 *mutants compared to WTs (Fig. 2H-J and Fig. S3H). These data correlate with a higher population of* $Slc13a5^{+}$ *neurons compared to* $Slc13a5^{+}$ *astrocytes in WT midbrain (Fig. 1G, H, and K), pointing to the dominance of neuronal expression of Slc13a5. We attempted to measure changes in astrocyte cell death (e.g.,* $CC3^{+}/GFAP^{+}$*) but the available antibody labels both the astrocyte soma and projections, making any co-label tricky to distinguish.*

UNIVERSITY OF CALGARY

To analyze calcium activity, we raised our knockout animals in the Tg(elavl3:Hsa.H2B-GCaMP6s) background, showing pan-neuronal GCaMP expression. Our previous and new calcium imaging data shows enhanced calcium activity in slc3a5 *mutants compared to WTs, as shown by all the neurons (elavl3$^+$) in the forebrain, midbrain and hindbrain (Fig. 6A-C, Fig. S11). We have included these new data on page 11.*

Previously, we showed that the midbrain size was reduced in our slc13a5 *mutants compared to WTs by measuring the brain width (Fig. 1T, 1V). We now show that midbrain volume also is reduced in* slc13a5 *mutants (Fig. S1K). These data can be found on pages 6 and 22.*

Our bioenergetics analyses shows that the slc13a5$^{-/-}$ *zebrafish exhibit a significant reduction in various parameters of neurometabolism, including basal respiration, ATP-linked respiration, total mitochondrial respiration and non-mitochondrial respiration (Fig. 4B-E). We speculate that intracellular citrate dysfunction is a possible cause of this imbalanced bioenergetics. Similarly, studies on cortical cell cultures generated from epileptic (low-Mg^{2+} model) rodent brains have demonstrated that prolonged seizure activity negatively affects mitochondrial bioenergetics and induces cell death (Kovac et al., Journal of Cell Science, 2012). Further, our group and others have also shown that mitochondrial bioenergetics is altered in various epileptic models of zebrafish (Kumar et al., eNeuro, 2016; Ibhazehiebo et al., Brain, 2018; Banerji et al., Brain Communications, 2021; Dogra et al., Epilepsia, 2023).*

Finally, regarding confounding factors when measuring whole zebrafish bioenergetics (versus just the brain): when we were establishing the zebrafish bioenergetics assays in the lab, we considered the potential 'contamination' of peripheral mitochondrial function to brain levels when measuring the whole fish. To test this, we assayed whole fish vs head alone vs whole body minus the head. To our surprise, the oxygen consumption rate data were the same when measuring whole body or severed heads, and were too low to detect reliably for the whole fish minus the heads (unpublished). We explain these data that the brain is an energy-demanding organ, requiring >25% of the body's glucose despite being just 2% of the overall weight (in humans). Thus, any contributions of the peripheral cells to the measured bioenergetic responses in the whole zebrafish is negligible at the resolution of the Seahorse Bioanalyzer.

A possible explanation for disturbed vglut2 and gad1b expression in our slc13a5 *mutants is that amongst various functions, citrate is also involved in glutamate/GABA synthesis and could plausibly alter the homeostasis of these neurotransmitters (Hertz, Frontiers in Endocrinology, 2013). Thus, the consequential excitatory/inhibitory (E/I) neuronal imbalance along with excessive calcium influx (via overactivated NMDA signaling) possibly contribute to higher seizure susceptibility in* slc13a5 *mutants. That said, we are not showing the direct role of*

UNIVERSITY OF CALGARY

intracellular citrate in glutamate/GABA synthesis and therefore, we have only mentioned that E/I neuronal balance is disturbed in our slc13a5 *mutants in the main text.*

We would also like to mention that in the revised manuscript, we have repeated *5a* and *gad1b* qPCR for the third biological replicates and have included updated qPCR graphs in Fig. S1C and Fig. 3B.

In summary, the Reviewers have made critical suggestions that improve our study. Thank you for choosing to review our paper and we look forward to receiving your advice on the suitability of our revised manuscript for *PLOS Biology*.

Thank you for your consideration.

Sincerely,

Deborah Kurrasch, PhD
Professor (on behalf of the authors)
