# Supplementary material for: Modulation of NMDA receptor signaling and zinc chelation prevent seizure-like events in a zebrafish model of SLC13A5 epilepsy
Source: PLoS Biol. 2025 Apr 10;23(4):e3002499. doi: 10.1371/journal.pbio.3002499 (PMC12047791; doi:10.1371/journal.pbio.3002499)
Supplement: Attachment — Submitted filename: Dogra et al_revision2_response to reviewers.pdf [file pbio.3002499.s034.pdf]

UNIVERSITY OF CALGARY

CUMMING SCHOOL OF MEDICINE

Department of Medical Genetics
Alberta Children's Hospital Research Institute
Hotchkiss Brain Institute
Room 2215/Health Science Centre
Foothills Campus, University of Calgary
3330 Hospital Drive NW
Calgary, Alberta Canada T2N 4N1

T +1.403.210.6713
F +1.403.210.8757
E kurrasch@ucalgary.ca

January 30, 2025

RE: Re-submission of Research Article "PBIOLOGY-D-24-00025R2" to *PLOS Biology*

Dear Dr Nonia Pariente,

We are pleased to resubmit the revised version of our manuscript "Modulation of NMDA receptor signaling and zinc chelation prevent seizure-like events in *slc13a5* loss-of-function zebrafish" by Deepika Dogra et al to *PLOS Biology*. We thank the Academic Editor and all the Reviewers for recognizing the improvements of our revised manuscript and for their continued careful considerations.

Here we address a few remaining Reviewer concerns either by providing more data analysis, revision of the text, and/or our responses below. Specifically, new data analysis is now included in Fig. 1, 5 and 6 and text (highlighted in track changes version). Please find below our responses (italicized) to each reviewer comment.

Below is a point-by-point response to each Reviewer's comment.

REVIEWER'S COMMENTS AND AUTHOR'S RESPONSES:

Reviewer: 2
Emre Yaksi (note, reviewer 2 has signed this review): I thank the authors for all additional work and clarifications. The revised manuscript has addressed my comments. My only remaining comment is that the text in some of the updated figures appears very small. I would highly recommend the authors for an update in some of their figure organization and text size.

We thank Reviewer 2 for acknowledging that we have addressed their comments. We have now updated the text size in all the figures and reorganized Fig. 6.

Reviewer: 3
Quynh Anh Nguyen (note, reviewer 3 has signed this review): Dogra et al. provide a comprehensive analysis of their zebrafish model of SLC13A5 deficiency and show the involvement of NMDA signaling and zinc chelation in the emergence of epilepsy-like

UNIVERSITY OF CALGARY

phenotypes. This new zebrafish model may be useful in future drug discovery studies to identify more effective treatments for the associated disease in humans.

1) The authors have addressed my concerns, especially providing more convincing data supporting the epilepsy-like seizure phenotype in their model. Although they still find that a majority of mutant animals do not display epileptic-like activity even with an additional more sensitive electrophysiological measure (Fig 3M), this model may still be useful for future drug screening studies. A remaining question is whether there is correlation between animals that display electrophysiological abnormal activity and the behaviors assayed, which will help provide a simple way to screen epileptic-like from non-epileptic like individuals in the mutants.

We thank Reviewer 3 for acknowledging that we have addressed their concerns and mentioning that we have provided "more convincing data". We appreciate their question about correlation between animals that display electrophysiological abnormal activity and the behaviors assayed. We have tried a similar experimental setup previously wherein we sorted slc13a5 *mutants for strong behavior phenotype, e.g., showing impaired response to acoustic startle (similar to Fig. 2A-C), followed by measuring extracellular field potentials (similar to Fig. 3F-I). Not all of those sorted mutants showed brain hyperexcitability in electrophysiological recordings. We speculate that this could be due to an incomplete penetrance of seizure-like phenotype in the* slc13a5 *mutants at larval stages, as discussed on page 15.*

2) One minor suggestion: please add a label to Fig 3M to distinguish it from Fig 3I, maybe a label stating NeuroProbe.

We appreciate this suggestion and have now mentioned "NeuroProbe" in Fig. 3M.

Reviewer: 4
The authors have substantiated the argument that the citrate transporter slc13a5 neuronal phenotype is caused by reduced extracellular zinc in two ways. 1) By measuring extracellular zinc (Palm-ZP1 staining) and 2) by showing that supplementing with ZnCl2 rescues many of the phenotypes. The ZnCl2 supplementation results are compelling and significantly strengthen the manuscript (Fig. 7K-P). However, the Palm-ZP1 quantitation (simply comparing levels of green fluorescence in WT vs mutant) is not done appropriately for several reasons: 1) the dye doesn't appear to penetrate far into the embryo, so the staining appears to be of superficial epithelial cells, not neurons. 2) the quantitation is simply of the Palm-ZP1 channel. The authors should compare the green:red ratio (Palm-ZP1:membrane stain) in WT and mutants.

UNIVERSITY OF CALGARY

We appreciate that Reviewer 4 has acknowledged that "the $ZnCl_2$ supplementation results are compelling and significantly strengthen the manuscript". Regarding the Palm-ZP1/CellMask Orange dyes experiment:

a) We agree with this observation and previously we tried to stain deeper in the brain to capture the neurons; however we could not incubate the animals with the dyes for a longer duration as the dye becomes internalized and loses its membrane-specific expression (as also explained by Radford et al., Chem Sci., 2014). Therefore, we were restricted to capturing only the membrane staining in epithelial cells of the brain region and study the effect of $ZnCl_2$ treatment accordingly.

b) We now provide the data comparing Palm-ZP1:CellMask Orange CTCF ratios in the plasma membrane in WT and mutants (Fig. 6Q, R). Our data shows that the mutants have a significant reduction in CTCF ratio compared to WTs (Fig. 6M, M', N, N' and Q). Further, $ZnCl_2$-treated WTs show a significant increase in CTCF ratio compared to untreated WTs (Fig. 6M, M', O, O' and Q), and $ZnCl_2$-treated mutants show a significant increase in CTCF ratio compared to untreated mutants (Fig. 6N, N', P, P' and Q). We now include these new data and explanation on pages 12, 21 and 32.

There remain several other concerns about the quality of the work that should also be addressed, though largely without further experiments:

1) The RNA in situs in Fig. 1A-F in no way show "restriction" of slc13a5 to the midbrain. Expression is widespread in the brain and the head periphery, with slightly stronger expression in the tectum (as well as other structures). The authors should revise this statement. Also, the fact that slc13a5 gene expression is detected in FACs-purified Elavl3-expressing cells is evidence that slc13a5 genes are expressed in neurons but it is not "evidence for neuron-specific expression". This statement should be revised.

We have now revised the expression statement to read: "perhaps slightly stronger expression in the midbrain" or "expression appears to get slightly stronger in the midbrain" on pages 5, 6, 15 and 29. While we agree that slc13a5 *expression is broadly expressed throughout the central nervous system at 25 hpf (Fig. 1A-B) and 3 dpf (Fig. 1C-D), we do feel the data supports a statement saying that* slc13a5 *expression might become more restricted to the midbrain by 5 dpf (Fig. 1E-F), especially for* slc13a5b *(Fig. 1F).*

Regarding the neuron-selective claim, we now state that "both the paralogs are highly expressed in the neurons compared to astrocytes" on pages 6 and 34, which is based on our data in Fig. 1G, H, K and Fig. S1E, F, G.

UNIVERSITY OF CALGARY

1’) The immunostaining of Slc13a5 is only reduced by 50% in double mutants (Fig. S1J). What is the antibody raised against? The methods say it was raised in rabbit but against which species' Slc13a5? N- or C-terminal peptides? If it was raised against a C-terminal peptide then it means that the remaining 50% of the staining detected in double mutants is non-specific. A western blot would be more quantitative and would determine if any full-length protein persists in mutants.

The SLC13A5 antibody used (https://www.thermofisher.com/antibody/product/SLC13A5-Antibody-Polyclonal/PA5-113058) is raised against the human immunogen “LQQMEATSAATEAGLELVDKGKAKELPGSQVIFEGPTLGQQEDQERKRL” (156-204AA) and is targeting the N-terminal domain. The corresponding 5a *and* 5b *mutations in our zebrafish mutants lie downstream of the immunogen target region. Therefore, this N-terminal epitope recognized by the antibody may remain intact in the mutant, even though the full-length protein is predicted to be truncated. Our immunostaining data showing 50% residual protein expression in mutants (Fig. S1H, I, J) falls in line with this assumption.*

Below is the snapshot from https://www.uniprot.org/uniprotkb/Q86YT5/entry#Q86YT5-1 showing human SLC13A5 immunogen target region of the used antibody (highlighted in blue) as well as corresponding zebrafish 5a *and* 5b *mutation sites (highlighted in red and green, respectively):*

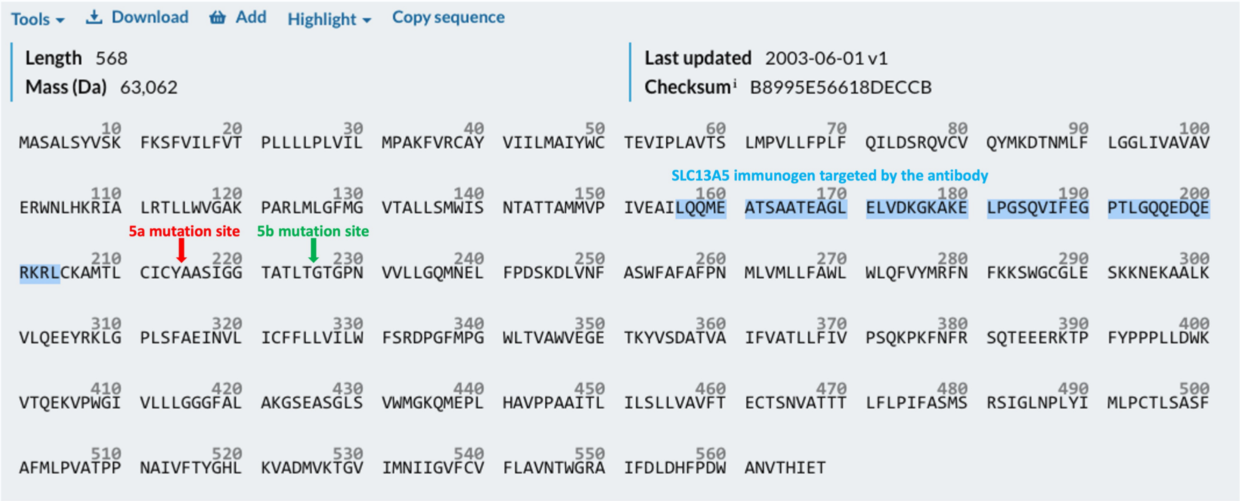

To address this reviewer’s concern, we did attempt a western blot on Slc13a5a/b double mutants and wild-type controls and the results were inconclusive due to technical issues. Western blots are not routine in my lab and the lead author is now on maternity leave, making it especially challenging to complete these studies. Given this situation, and although we

UNIVERSITY OF CALGARY

recognize the rationale for this reviewer's request, we counter-argue that these data would not change the overall interpretation of our findings. Specifically:

a) *The antibody does not differentiate between Slc13a5a and Slc13a5b isoforms, making any differences in the relative proportions of the bands on a Western blot inconclusive*
b) *The antibody does not bind the C-term of the protein, thereby alleviating any confounding issues of non-selectivity, the primary concern*
c) *The immunohistochemistry staining shows a large reduction in Slc13a5a/b+ cells in the brain of double mutants, with only a few cells labelled within this region of interest (Fig. S1H-I), consistent with a decrease of protein expression*
d) *Further, when Slc13a5a/b protein levels are quantified within a single cell (Fig S1J), double mutants show a 50% reduction compared to wild-types, suggestive of nonsense-mediated decay or loss of stability of the truncated protein arising from a loss-of-function mutation*
e) *The* slc13a5a *and* slc13a5b *transcripts are reduced to 25% in the double knockouts (Fig. S1C-D), also consistent with nonsense-mediated mRNA decay and a reduction in the steady-state level of the transcript*

Combined, multiple lines of evidence point to a loss of protein that is consistent with the numerous findings throughout the text showing a loss-of-function phenotype.

2) The immunostaining in Fig. 1G-J is undecipherable. Is the Slc13a5 the green speckles? Given that 50% of the staining may be non-specific (my point above), these images do not add any valuable information. Also, there is no mention of the co-staining with vGlut1 and Gad67 (Fig. 1I,J) and since these images are uninterpretable, these panels should be removed. Note: the legend says that there is Hoechst staining, but it is not shown in the image.

Slc13a5a/b are expressed on the plasma membrane, which is exactly where we see strong immunostaining. To make the expression pattern more pronounced, we have now added insets in Fig. 1G-J to show representative cells co-labeled with Slc13a5 and neuronal and astrocyte markers. We have added text discussing the co-expression of Slc13a5 with vGlut1 and Gad67 (Fig. 1I, J) on page 5. We have also corrected the figure legend of Fig. 1 and removed "Hoechst".

3) The authors have added RNA-Seq comparing WT, single and double mutants with the conclusion that slc13a5a contributes more to the double mutant phenotype than slc13a5b. However apart from the PCA plot, which is an abstraction, it is impossible to assess this assertion because the heat maps in Fig. 5 and Fig. S7 don't include all four genotypes. There should be counts for all the genes shown in all of the genotypes, so there is no reason to split

out the slc13a5b data from the slc13a5a and double mutant data. All the affected genes should be shown for all four genotypes in the same heat map.

To support our PCA plot data, we now utilized a count matrix of all genes to calculate sample-wise distances and have visualized the results using a heatmap. The resulting distance matrix (shown below) indicates that the double mutant samples are more closely related to the 5a *mutant samples than the* 5b *mutant samples.*

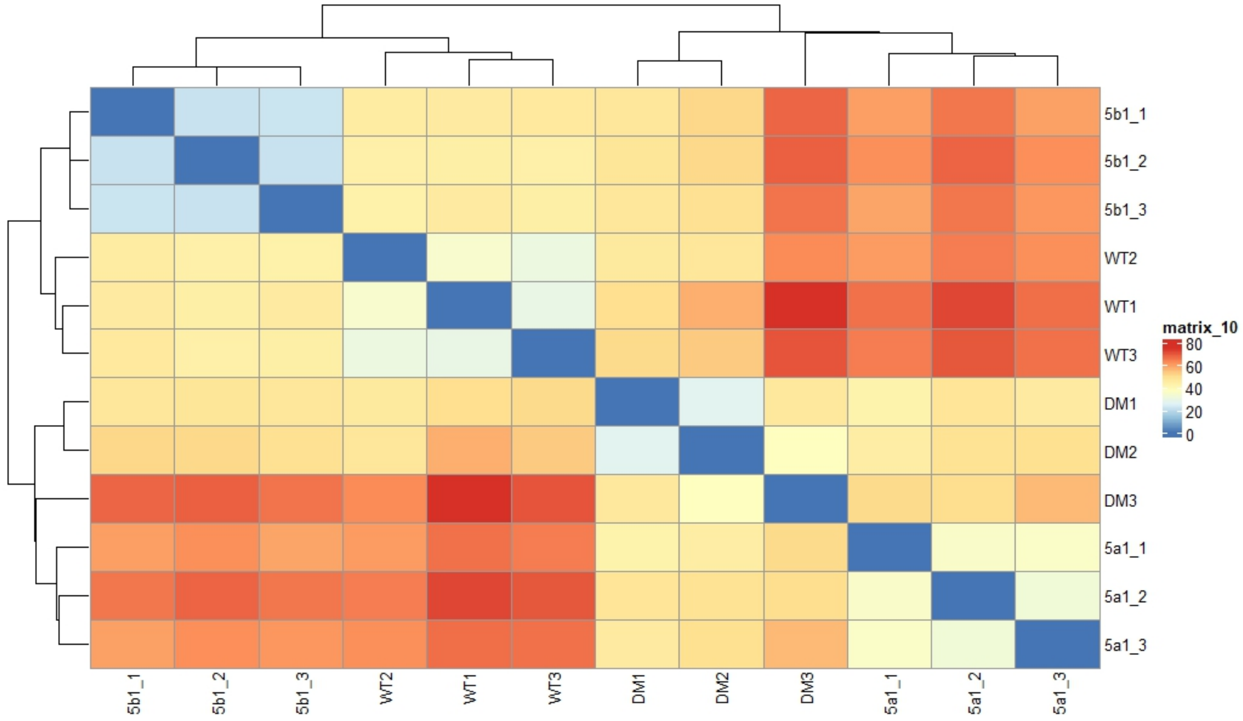

Further, as the reviewer suggested, we now replaced the separate heatmaps in Fig.5 with two new heatmaps combining data from all four genotypes. The description of the new heatmaps is mentioned on pages 10, 31, and 32.

4) Fig. 2F,G shows significant reduction in the number of neurons in the tectum, but Fig. 6 H,I, using a different marker of mature neurons, NeuN, shows a significant increase in the number of neurons (and a corresponding increase in grin1 expression). How are these contradictory results reconciled?

Our previous Fig. 6H was misleading as it was from a region of WT midbrain with faint NeuN expression. We have now replaced Fig. 6H with a better representative image showing that there are no changes in neuron numbers amongst WT and mutants.

UNIVERSITY OF CALGARY

5) The mutant generation and genotyping section of the methods says "WTs, single homozygous (5a-/- and 5b-/-) and double homozygous (5a-/-;5b-/-) larvae were used for the experiments (by breeding heterozygous adults)". This means that for each double mutant "n" in the manuscript, of which there are a lot, ~16 animals would have been assayed and subsequently genotyped. Is this really the case? If so, there should be vastly more WT and single mutant n's than double mutant n's but this doesn't appear to be the case. If, in fact, homozygous mutants were bred together for experiments, what was the source of WT controls? How were the experimenters blinded to the genotypes of the animals they were assaying?

As stated in the methods, we only used heterozygous adults for breeding so yes, we did generate more wild-types and single mutants than we ended up using in the assays. Originally, our strategy was to focus this study on double mutants vs. WTs and therefore we collected higher numbers of double mutants. Later in the project, we realized the single mutants had interesting phenotypes as well, so we collected those samples also from independent heterozygote crosses. WTs used in our experiments were obtained alongside the breeding of these heterozygous adults. All experiments were performed blinded, with larvae genotyped after the completion of the experiment.

6) The methods section for electrophysiology says that animals were anesthetized with Tricaine and bungarotoxin "to anesthetize and paralyze" the larvae and that the anesthetic was added to the recording bath. How then do the animals generate the twitches quantified in Fig. 3? It isn't valid to quantify body movement under paralyzing conditions, since any differences between WT and mutants may reflect a resistance to anaesthetic/paralytic in mutants rather than an intrinsic increased excitability.

We initially tested various doses of tricaine and alpha-bungarotoxin to fully anesthetize and paralyze the larvae. Interestingly, we observed that the mutants were more sensitive to the treatment. The minimum dosage required to fully anesthetize and paralyze all WT larvae proved lethal to most mutants, resulting in a flatline in the electrophysiological recordings. As a result, we adopted the dosage detailed in the Methods section. This dose was sufficient to paralyze both WT and mutant larvae for the majority of the recording period. However, we occasionally observed a single spike in the recordings, accompanied by a visible twitch on the camera. Notably, through extensive preliminary testing, the repeated twitches described in the results section were observed exclusively in mutants and never in WTs, irrespective of the dosage used. This finding gave us confidence that this behavior, as captured indirectly through electrophysiological recordings, was a distinct characteristic of the mutants.

7) Statistics: Two different symbols are used to indicate $P<0.01$: ** and ##. What is the difference between them? Please clarify.

UNIVERSITY OF CALGARY

*Two symbols for p-value were used whenever we were comparing two different groups (for e.g., # for WT vs. others, * for mutant vs. others). We have now changed all symbols to * to keep it uniform throughout the figures.*

In summary, we would like to thank the Reviewers for their critical and insightful suggestions that significantly improved our study. We look forward to receiving your advice on the suitability of our revised manuscript for *PLOS Biology*.

Thank you for your consideration.

Sincerely,

Deborah Kurrasch, PhD
Professor (on behalf of the authors)
